# Tracing Evolving Networks Using Tensor Factorizations vs. ICA-Based Approaches

**DOI:** 10.3389/fnins.2022.861402

**Published:** 2022-04-25

**Authors:** Evrim Acar, Marie Roald, Khondoker M. Hossain, Vince D. Calhoun, Tülay Adali

**Affiliations:** ^1^Simula Metropolitan Center for Digital Engineering, Oslo, Norway; ^2^Oslo Metropolitan University, Oslo, Norway; ^3^Department of Computer Science and Electrical Engineering, University of Maryland Baltimore County, Baltimore, MD, United States; ^4^Department of Psychology, Georgia State University, Atlanta, GA, United States

**Keywords:** PARAFAC2, independent vector analysis (IVA), independent component analysis (ICA), tensor factorizations, spatial dynamics, evolving networks, time-evolving data

## Abstract

Analysis of time-evolving data is crucial to understand the functioning of dynamic systems such as the brain. For instance, analysis of functional magnetic resonance imaging (fMRI) data collected during a task may reveal spatial regions of interest, and how they evolve during the task. However, capturing underlying spatial patterns as well as their change in time is challenging. The traditional approach in fMRI data analysis is to assume that underlying spatial regions of interest are static. In this article, using fractional amplitude of low-frequency fluctuations (fALFF) as an effective way to summarize the variability in fMRI data collected during a task, we arrange time-evolving fMRI data as a *subjects* by *voxels* by *time windows* tensor, and analyze the tensor using a tensor factorization-based approach called a PARAFAC2 model to reveal spatial dynamics. The PARAFAC2 model jointly analyzes data from multiple time windows revealing subject-mode patterns, evolving spatial regions (also referred to as networks) and temporal patterns. We compare the PARAFAC2 model with matrix factorization-based approaches relying on independent components, namely, joint independent component analysis (ICA) and independent vector analysis (IVA), commonly used in neuroimaging data analysis. We assess the performance of the methods in terms of capturing evolving networks through extensive numerical experiments demonstrating their modeling assumptions. In particular, we show that (i) PARAFAC2 provides a compact representation in all modes, i.e., *subjects, time*, and *voxels*, revealing temporal patterns as well as evolving spatial networks, (ii) joint ICA is as effective as PARAFAC2 in terms of revealing evolving networks but does not reveal temporal patterns, (iii) IVA's performance depends on sample size, data distribution and covariance structure of underlying networks. When these assumptions are satisfied, IVA is as accurate as the other methods, (iv) when subject-mode patterns differ from one time window to another, IVA is the most accurate. Furthermore, we analyze real fMRI data collected during a sensory motor task, and demonstrate that a component indicating statistically significant group difference between patients with schizophrenia and healthy controls is captured, which includes primary and secondary motor regions, cerebellum, and temporal lobe, revealing a meaningful spatial map and its temporal change.

## 1. Introduction

Time-evolving data analysis is crucial in terms of understanding complex dynamic systems such as the brain. Various neuroimaging techniques such as functional magnetic resonance imaging (fMRI) and electroencephalography (EEG) are used to collect temporal data in order to understand how the brain functions. The analysis of such temporal data may capture the underlying patterns as well as their temporal evolution revealing the underlying mechanisms, and how those differ across different groups of people, e.g., healthy controls vs. patients. For instance, the relation between dynamic functional connectivity and various disorders such as schizophrenia, autism, and Alzheimer's disease has been studied with the goal of finding biomarkers (Preti et al., 2017).

Dynamic functional connectivity (also referred to as time-varying functional connectivity) has been an important topic of research to study brain function (Chang and Glover, 2010; Hutchison et al., 2013a; Calhoun et al., 2014; Preti et al., 2017; Lurie et al., 2020). The most commonly used approach for dynamic functional connectivity analysis is the sliding window-based method (Sakoglu et al., 2010), where correlations between time courses corresponding to different spatial regions of interest are used to construct a connectivity matrix for each time window. Functional connectivity patterns from each window are then analyzed using various methods such as graph mining to understand the change in time. Often there is the simplifying assumption that spatial regions of interest are static, and it is only the connectivity between those static spatial regions that changes in time. On the other hand, it has been previously shown that there are changes in spatial regions as well even in the resting state (during awake as well as anesthetized states; Kiviniemi et al., 2011; Hutchison et al., 2013b; Ma et al., 2014).

Our focus here is on the analysis of fMRI signals collected during a task with the goal of revealing spatial regions of interest as well as the temporal evolution of those regions (i.e., spatial dynamics; Iraji et al., 2020). Low-rank data approximations [matrix factorizations as well as tensor factorizations (Acar and Yener, 2009; Kolda and Bader, 2009; Comon, 2014), i.e., extensions of matrix factorizations to higher-order data] have proved useful in terms of revealing the underlying patterns in complex data in many fields including neuroscience, e.g., revealing spatial regions of interest/networks (McKeown et al., 1998; Bai et al., 2017). Recently, various matrix factorization-based approaches including independent component analysis (ICA) and principal component analysis (PCA) have been studied in terms of tracking functional connectivity by arranging magnetoencephalography (MEG) signals as a *connectivity* by *time* matrix and factorizing the matrix into temporal patterns and connectivity patterns revealing brain networks (Tabbal et al., 2021) with the assumption that networks relying on predefined regions of interests stay the same in time. However, capturing patterns evolving in time from dynamic data such as evolving networks, evolving spatial regions or evolving communities remains a challenging data mining problem (Rossetti and Cazabet, 2018). Previously, ICA (Comon, 1994) was used together with a sliding time window-based approach to study the changes in spatial maps, focusing on the changes within default mode networks (DMN) in time in the resting state (Kiviniemi et al., 2011). Similarly, Ma et al. (2014) used independent vector analysis (IVA) (Kim et al., 2006; Anderson et al., 2012), i.e., an extension of ICA to multiple datasets, to find time-varying brain networks during the resting state. These studies focus on resting-state dynamics, and also are limited due to either the focus on a single network (Kiviniemi et al., 2011), or not revealing compact patterns in the *time* mode explicitly (Kiviniemi et al., 2011; Ma et al., 2014).

**Figure 1:**
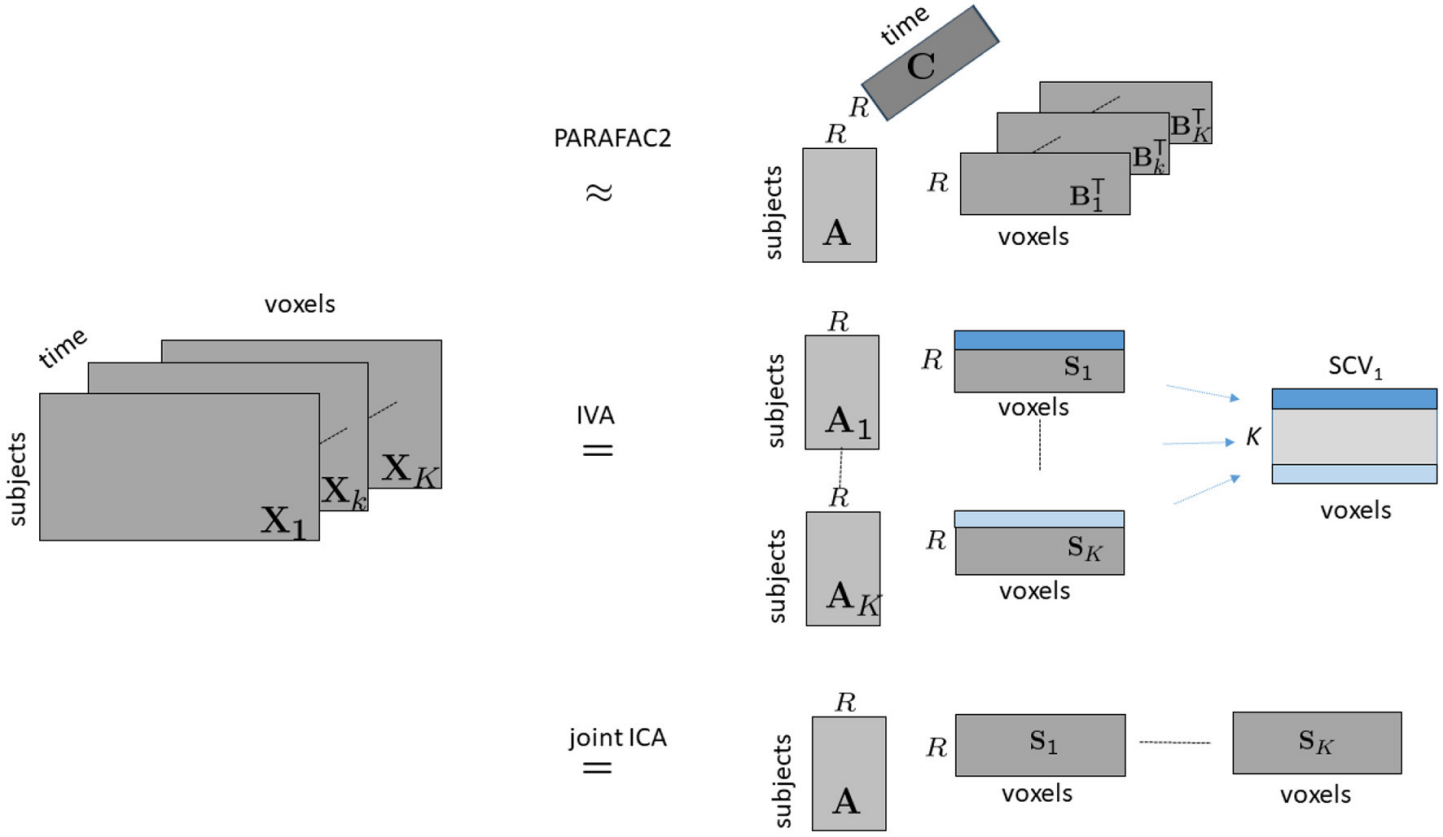
Illustration of modeling time-evolving data in the form of a *subjects* by *voxels* by *time* tensor using PARAFAC2, IVA and joint ICA. Following the notation in the literature on ICA/IVA, we use S_*k*_, for *k* = 1, …, *K*, to denote the factor matrix in the *voxels* mode for joint ICA and IVA, where ${\text{S}}_{k}={\text{B}}_{k}^{T}$.

As higher-order tensors are natural data representations for temporal data, with one of the modes representing time, in this article, through the use of fractional amplitude of low-frequency fluctuations (fALFF), we arrange fMRI data collected during a task as a third-order tensor with modes: *subjects, voxels*, and *time windows*, and use a tensor factorization method called the PARAFAC2 model (Harshman, 1972; Kiers et al., 1999), which compactly summarizes the dynamic data by revealing the underlying networks (spatial regions of interest), their change in time as well as temporal patterns (see Figure 1). More specifically, we use the PARAFAC2 model to jointly factorize multiple matrices in the form of *subjects* by *voxels* matrices, X_*k*_ for *k* = 1, …, *K*, corresponding to different time windows, coupled in the *subjects* mode, where *K* denotes the number of time windows. The PARAFAC2 model summarizes the data using low-rank patterns in the *subjects, voxels*, and *time windows* modes, and the patterns in the *voxels* mode change from one window to another revealing the evolving patterns. Patterns in the *time windows* mode correspond to temporal patterns, and patterns in the *subjects* mode can be used to explore differences between healthy controls and patients, or for patient stratification.

While the use of tensor factorizations in neuroimaging signal analysis has been widespread (Cong et al., 2015; Hunyadi et al., 2017), to the best of our knowledge, their potential for revealing spatial dynamics has not been explored. Neuroimaging data, e.g., EEG (Miwakeichi et al., 2004), fMRI (Andersen and Rayens, 2004), MEG (Becker et al., 2012), local field potential (LFP) (Geddes et al., 2020) signals, can be represented as higher-order tensors. Tensor methods can reveal interpretable patterns from such complex data disentangling different sources as a result of their uniqueness properties (Kolda and Bader, 2009), avoiding additional constraints on the underlying patterns such as orthogonality or statistical independence. For instance, multi-channel EEG signals have been arranged as a *time* by *frequency* by *channels* tensor, and analyzed using the CANDECOMP/PARAFAC (CP) (Hitchcock, 1927; Carroll and Chang, 1970; Harshman, 1970) tensor model revealing spatial, spectral and temporal signatures of brain activities (Miwakeichi et al., 2004; Acar et al., 2007; De Vos et al., 2007). In the case of multiple subjects/conditions, the CP model has similarly shown promising performance in terms of revealing the underlying patterns (Möcks, 1988; Mørup et al., 2006). The higher-order structure of fMRI signals has also been studied using tensor methods, e.g., by arranging fMRI signals as a *trials* by *voxels* by *time* tensor, and analyzing the tensor using the CP model (Andersen and Rayens, 2004), or analyzing multi-subject fMRI data in the form of a *subjects* by *voxels* by *time* tensor using tensor probabilistic independent component analysis (PICA) (Beckmann and Smith, 2005). Such CP-based models, for instance when analyzing multi-subject fMRI data, extract subject-mode patterns, spatial patterns as well as temporal patterns with the modeling assumption that subject-mode patterns and spatial patterns are the same in all time slices (up to a scaling) (Beckmann and Smith, 2005); therefore, not accounting for spatial dynamics. While brain images are unfolded and treated as vectors of voxels resulting in third-order tensors in these studies, higher-order fMRI data as fourth and fifth-order tensors have also been studied by preserving the spatial structure (Chatzichristos et al., 2019). Recently, the PARAFAC2 model, which is more flexible than the CP model, has been used to study functional connectivity using multi-subject fMRI signals by letting the temporal patterns change across subjects (Madsen et al., 2017; Helwig and Snodgress, 2019) assuming common (and static) spatial patterns for all subjects. In the context of dynamic functional connectivity, Zhu et al. (2019, 2020) have arranged MEG signals as a *time* by *frequency* by *connectivity* tensor, where connectivities rely on predefined anatomical regions, and analyzed the tensor using a CP model to reveal connectivity factors showing functional networks. However, none of these studies accounts for evolving spatial patterns or evolving networks but rather all rely on static spatial patterns/networks.

This article is an extended version of our preliminary study (Roald et al., 2020), where we demonstrated the promise of the PARAFAC2 model in terms of revealing evolving networks using simulations and real task fMRI data analysis. In this work, we provide an extensive study comparing the performance of the PARAFAC2 model with ICA-based approaches, in particular, joint ICA (Calhoun et al., 2006) and IVA, in terms of capturing evolving networks using different simulation set-ups demonstrating the effect of sample size, similar or overlapping networks, and differences in subject-mode patterns across time windows. Both joint ICA and IVA are extensions of ICA to multiple datasets but rely on different modeling assumptions. Their use in the comparison is attractive as IVA is less constrained than PARAFAC2 by letting subject-mode patterns change from one time window to another and has been previously used for capturing spatial dynamics (Ma et al., 2014) while joint ICA is similar to PARAFAC2 in the way it models the subject-mode patterns but differs in terms of constraints imposed on the evolving networks. We also use our observations from simulations to guide our analysis of multi-site multi-subject fMRI data (Gollub et al., 2013) collected during a sensory motor task. While our preliminary results (Roald et al., 2020) focused on the analysis of a subset of the data from several sites using a PARAFAC2 model, in this article, we use the data from all sites, and study the application of all three methods to this task-related fMRI data, and compare their performances. Our experiments demonstrate that (i) PARAFAC2 provides a compact representation revealing temporal patterns and evolving spatial networks accurately, (ii) joint ICA is as effective as PARAFAC2 in terms of revealing evolving networks but does not reveal temporal patterns explicitly, (iii) IVA's performance depends on sample size. We also show its assumptions on data distribution and covariance structure of underlying networks in the Supplementary Material. When these assumptions are fulfilled, IVA is as accurate as the other methods in terms of capturing underlying networks, and in addition, (iv) IVA can reveal evolving networks accurately when subject-mode patterns differ across time windows, (v) in real fMRI data analysis, a meaningful component indicating statistically significant group difference between patients with schizophrenia and healthy controls is captured by all methods revealing a spatial network of potential interest as well as its change in time. Guided by the simulations, we discuss the accuracy of estimated components and their significance in terms of group difference.

## 2. Materials and Methods

### 2.1. Background

We first briefly discuss modeling assumptions of the three methods we focus on, namely, PARAFAC2, IVA, and joint ICA.

#### 2.1.1. PARAFAC2

Given a third order tensor, X ∈ ℝ^*I*×*J*×*K*^, the PARAFAC2 model represents each slice, ${\text{X}}_{k}\in {\mathbb{R}}^{I\times J}$, as follows:


(1) $$\begin{array}{l} {\text{X}}_{k}\approx \text{A}\text{diag}\left(\text{c}\left(k,:\right)\right){\text{B}}_{k}^{T}, \end{array}$$

where A ∈ ℝ^*I*×*R*^, ${\text{B}}_{k}\in {\mathbb{R}}^{J\times R}$, *R* is the number of components, and diag(c(*k*, :)) is a diagonal matrix with entries of the *k*th row of C ∈ ℝ^*K*×*R*^ on the diagonal. Additionally, B_*k*_-matrices satisfy the constant cross product constraint, ${\text{B}}_{k_{1}}^{T}{\text{B}}_{k_{1}}={\text{B}}_{k_{2}}^{T}{\text{B}}_{k_{2}}$ for all 1 ≤ *k*_1_, *k*_2_ ≤ *K*. The PARAFAC2 model reveals unique factors (up to scaling and permutation ambiguities) as long as there are enough slices (*K*) (see Kiers et al., 1999 for a detailed discussion on uniqueness conditions of PARAFAC2). The traditional algorithmic approach to fit the model is by solving the following optimization problem using an alternating least squares (ALS)—based algorithm (Kiers et al., 1999):


(2) $$\begin{array}{l} \underset{\text{A},{\left\{{\text{B}}_{k}\right\}}_{k\leq K},\text{C}}{\min}\overset{K}{\underset{k=1}{\sum }}||{\text{X}}_{k}-\text{A}\text{diag}\left(\text{c}\left(k,:\right)\right){\text{B}}_{k}^{T}|{|}_{F}^{2}, \end{array}$$

where B_*k*_ = P_*k*_B, and ${\text{P}}_{k}^{T}{\text{P}}_{k}=\text{I}$ so that the constant cross product constraint is implicitly satisfied; I ∈ ℝ^*R*×*R*^ denotes the identity matrix, B ∈ ℝ^*R*×*R*^ is common for all B_*k*_, *k* = 1, …, *K*, and ||·||_*F*_ denotes the Frobenius norm. Note that there may be an additional sign ambiguity in PARAFAC2, where each entry in diag(c(*k*, :)) may flip sign arbitrarily (Harshman, 1972), and one possible solution to fix that ambiguity is to impose non-negativity constraints on matrix C (Harshman, 1972; Kiers et al., 1999).

If X_*k*_s correspond to *subjects* by *voxels* matrices at different time windows, PARAFAC2 reveals subject-mode patterns (A) that are constant in time, and time-mode patterns (C) shared between subjects. The number of components *R* corresponds to the number of patterns. The PARAFAC2 model also reveals spatial networks (B_*k*_) that are shared between subjects but may evolve with time (as shown in Figure 1). This is a more flexible modeling approach than the most commonly used CP tensor model, which represents each slice ${\text{X}}_{k}\in {\mathbb{R}}^{I\times J}$ of a third-order tensor, X ∈ ℝ^*I*×*J*×*K*^, as follows:


(3) $$\begin{array}{l} {\text{X}}_{k}\approx \text{A}\text{diag}\left(\text{c}\left(k,:\right)\right){\text{B}}^{T}, \end{array}$$

where B ∈ ℝ^*J*×*R*^ representing spatial networks are assumed to be the same (more precisely, they can only change up to a scalar) across different time windows. We have previously demonstrated that the CP model fails to reveal underlying networks accurately when analyzing data generated using evolving network patterns while PARAFAC2 achieves to reveal the underlying networks as well as their change in time (Roald et al., 2020). Similar to CP, tensor PICA (Beckmann and Smith, 2005) also relies on (3), with the additional constraint that columns of B, e.g., spatial networks, are statistically independent. Therefore, both CP and PICA assume that spatial networks are the same across time slices, i.e., they do not account for evolving spatial networks as the PARAFAC2 model does by introducing B_*k*_s that allow for spatial networks to change more than a scalar factor.

Determining the number of components in tensor factorizations is a challenging task. There are various diagnostic approaches that can potentially be used to determine the number of components for PARAFAC2 such as the core consistency diagnostic (Kamstrup-Nielsen et al., 2012), and split-half analysis (Harshman and De Sarbo, 1984); both of which are also used to determine the number of components when fitting a CP model. However, a good practice is to use such diagnostic methods while taking into account also the factors and residuals (Bro and Kiers, 2003; Kamstrup-Nielsen et al., 2012). Note that uniqueness conditions of the PARAFAC2 model may also limit the number of components. For instance, one of the uniqueness conditions indicates that $K\geq \frac{R\left(R+1\right)\left(R+2\right)\left(R+3\right)}{24}$, where *K* and *R* denote the number of slices and components, respectively. This is a sufficient (not necessary) condition for the uniqueness of the model, and other studies have reported uniqueness using much fewer slices in practice (Kiers et al., 1999).

While the use of PARAFAC2 is not as widespread as the CP model, it has shown promising performance in applications from different disciplines, e.g., chemometrics (Bro et al., 1999), text mining (Chew et al., 2007), electronic health record analysis (Afshar et al., 2018; Yin et al., 2020), and neuroimaging data analysis (Madsen et al., 2017; Helwig and Snodgress, 2019). The use of PARAFAC2 in time-evolving data analysis, on the other hand, has been limited, where the model is used to analyze temporal data by letting the patterns change across subjects (Timmerman and Kiers, 2003; Madsen et al., 2017), or across channels (Weis et al., 2010) but not revealing dynamic networks.

#### 2.1.2. Independent Vector Analysis (IVA)

Similar to PARAFAC2, IVA also jointly analyzes multiple matrices. However, unlike PARAFAC2, IVA (Kim et al., 2006; Anderson et al., 2012; Adali et al., 2014) extracts statistically independent components (sources) from each matrix while taking into account the dependence across the datasets. In many applications using ICA and IVA, reducing the dimensionality of the observed dataset prior to analysis, i.e., identifying a signal subspace where to perform the decomposition enables better generalization performance decreasing the effect of noise and artifacts, also improving stability of the decompositions (see, e.g., Li et al., 2007). This is typically achieved using a PCA step, where the dimensionality of the observation matrix X ∈ ℝ^*I*×*J*^ is reduced from X ∈ ℝ^*I*×*J*^ to $\bar{\text{X}}\in {\mathbb{R}}^{R\times J}$ where *R* ≤ *I*.

Given *K* dimension-reduced observation matrices ${\bar{\text{X}}}_{k}\in {\mathbb{R}}^{R\times J}$, for *k* = 1, …, *K*, IVA models each dataset as a linear mixture of *R* independent sources:


(4) $$\begin{array}{l} {\bar{\text{X}}}_{k}={\bar{\text{A}}}_{k}{\text{S}}_{k}, \end{array}$$

where ${\bar{\text{A}}}_{k}\in {\mathbb{R}}^{R\times R}$ corresponds to the nonsingular mixing matrix, and ${\text{S}}_{k}\in {\mathbb{R}}^{R\times J}$ denotes the samples of independent sources for the *k*th matrix^1^. Corresponding components in S_*k*_ matrices form, the source component vectors (SCV), which are shown as matrices assuming a given set of observations, in Figure 1. IVA estimates the demixing matrices W_*k*_ to recover source estimates through ${\text{Y}}_{k}={\text{W}}_{k}{\bar{\text{X}}}_{k}$ by maximizing independence across the SCVs through mutual information minimization (Adali et al., 2014), which can be shown to be equivalent to maximum likelihood (ML) estimation. The estimated mixing matrices are then back reconstructed in the original dimensionality as explained in Jia et al. (2021), which implies that we effectively have the generative model shown in Figure 1. Thus, here, we show the IVA and joint ICA models in the original dimensionality to allow easier comparison with the PARAFAC2 model, which does not involve such a dimension reduction stage.

By modeling the multivariate probability density function (pdf) of an SCV, IVA takes the statistical dependence across the datasets into account, and depending on the chosen pdf, either, only second-order statistics (SOS), or all-order statistical information can be taken into account. In this work, we use IVA-L-SOS where a full multivariate Laplacian pdf model, also computing the scatter matrices is used (Bhinge et al., 2019b), hence taking all-order statistics into account. As fMRI sources tend to be super-Gaussian in nature (Correa et al., 2007; Calhoun et al., 2013), IVA-L-SOS provides a good match to their properties.

It can be shown that IVA has very general conditions for the identifiability of the model. For the case we consider where sample dependence is not taken into account and all-order statistics are used, the model is uniquely identifiable as long as the covariance matrices R_*l*_ and R_*m*_ of any two SCVs, *l* and *m*, are multivariate Gaussian and do not satisfy R_*l*_ = DR_*m*_D where D is any full rank diagonal matrix (Anderson et al., 2012; Adali et al., 2014). When only a subset of Gaussian components satisfy the equality, a subspace of their mixtures is identified and not the specific Gaussian components.

When X_*k*_ matrices represent *subjects* by *voxels* matrices at different time windows, IVA captures subject-mode patterns (A_*k*_) for each time window, and spatial components/networks, S_*k*_, changing from one window to another. Rows of S_*k*_s are related across the time windows through SCVs in such a way that mutual information within each SCV, i.e., statistical dependence, is maximized. Hence, the desire to capture the relationship among components in different S_*k*_s makes IVA another candidate approach for capturing evolving networks. IVA has been previously used to study dynamics in multi-subject resting-state fMRI data (Ma et al., 2014; Bhinge et al., 2019a,b; Long et al., 2021). For instance, Ma et al. (2014) arranges the data in a specific time window from a subject as a matrix in the form of *time samples* by *voxels*. Subject-specific temporal and spatial patterns are identified on a per window basis. This allows study of both temporal and spatial patterns of dynamics, however, the complexity of the model grows with the number of time windows, which negatively affects the performance of IVA (Long et al., 2020). Our approach in this article makes use of the synchrony across subjects in the task, and decreases the dimensionality of the problem by collapsing the time dimension through the use of fALFF as features for each time window (see section 2.2.4 for more details). As such, this provides an attractive formulation for dynamic analysis using IVA (Hossain et al., 2022).

#### 2.1.3. Joint Independent Component Analysis

Another approach to jointly analyze multiple matrices, ${\text{X}}_{k}\in {\mathbb{R}}^{I\times J}$, for *k* = 1, …, *K*, is to concatenate different time windows, and then analyze the constructed matrix using an ICA model, which is called the joint ICA (Calhoun et al., 2006) method. We again write the model using dimension-reduced observations matrices ${\bar{\text{X}}}_{k}\in {\mathbb{R}}^{R\times J}$, such that we have


(5) $$\begin{array}{l} \left[{\bar{\text{X}}}_{1}\;{\bar{\text{X}}}_{2}\ldots {\bar{\text{X}}}_{K}\right]=\bar{\text{A}}\text{S}, \end{array}$$

where $\bar{\text{A}}\in {\mathbb{R}}^{R\times R}$ corresponds to the non-singular mixing matrix that is common for all time windows, and S ∈ ℝ^*R*×*JK*^ represents the source signals corresponding to the spatial networks concatenated in time. Source signals, i.e., rows of S, are assumed to be statistically independent. ICA reveals unique components and mixing matrices, up to scaling and permutation ambiguities (Comon, 1994). When only non-Gaussianity is used as signal diversity ignoring sample dependence, any signal except multiple Gaussians can be identified with the model (Cardoso, 2001; Adali et al., 2014). Among various algorithmic approaches, in our experiments, we use an ICA algorithm based on entropy bound minimization (ICA-EBM), which uses a flexible pdf model, and hence can effectively model sources from a rich class of distributions (Li and Adali, 2010).

Again in Figure 1, we show the model for joint ICA following back-reconstruction where X_*k*_ and A are brought to their original dimensionality following ICA. Then, with X_*k*_ matrices corresponding to *subjects* by *voxels* matrices at different time windows, joint ICA reveals subject-mode patterns, A, shared by all time windows, and different spatial components/networks for each time window, i.e., S = [S_1_ S_2_…S_*K*_].

For ICA and IVA, a common approach for determining the number of components is the use of information theoretic criteria (ITC) such as minimum description length based on a PCA formulation (Wax and Kailath, 1985). ITC are based on a likelihood formulation based on the multivariate Gaussian assumption for the mixtures (a good match to the ICA/IVA mixing model). Since fMRI data exhibits sample correlation, usually a corrected version of the criteria are commonly employed as in Fu et al. (2014).

#### 2.1.4. PARAFAC2 vs. IVA vs. Joint ICA

Here we recap the modeling assumptions of different methods, specifically focusing on our application of interest, where X_*k*_ matrices correspond to *subjects* by *voxels* matrices at different time windows *k*.

- *Subject-mode patterns* (i.e., A_*k*_): Joint ICA extracts patterns that are the same in each time window, i.e., A_*k*_ = A, for *k* = 1, …, *K*; PARAFAC2 reveals patterns that are the same up to a scaling in each time window, i.e., A_*k*_ = Adiag(c(*k*, :)) while IVA is the most flexible one with no constraints on A_*k*_s.
- *Spatial components* (i.e., ${\text{B}}_{k}^{T}$ or S_*k*_): In IVA, S_*k*_s are more constrained than PARAFAC2 and joint ICA. In the IVA model, in each S_*k*_, the components are statistically independent, and across different S_*k*_s, the components are related through the SCVs; in PARAFAC2, there is the constant cross product constraint, ${\text{B}}_{k_{1}}^{T}{\text{B}}_{k_{1}}={\text{B}}_{k_{2}}^{T}{\text{B}}_{k_{2}}$ for all 1 ≤ *k*_1_, *k*_2_ ≤ *K*, while in joint ICA, there is the assumption of statistically independent components, and no relation between different S_*k*_s except that the sources in corresponding rows are all assumed to come from the same distribution.
- *Temporal components* (i.e., C): Among the three methods, the PARAFAC2 model is the most compact and reveals *temporal patterns* in addition to subject-mode and voxel-mode patterns while joint ICA and IVA only reveal patterns in *subjects* and *voxels* modes. In joint ICA and IVA, further postprocessing, possibly with additional assumptions, is needed to reveal temporal patterns.

### 2.2. Experiments

By using both real and simulated time-evolving data, we demonstrate the performance of PARAFAC2, IVA, and joint ICA in terms of capturing evolving networks. In simulations, we assess the performance of the methods in terms of how well they reveal the underlying ground truth. In the analysis of multi-subject fMRI data collected during a sensory motor task from patients with schizophrenia and healthy controls, the performance of the methods is assessed in terms of revealing meaningful components indicating statistically significant group differences.

#### 2.2.1. Implementation Details

All experiments were performed using MATLAB. Both simulated and real data are in the form of third-order tensors consisting of *K* frontal slices. The PARAFAC2 model is fit using the implementation in the PLS_Toolbox 8.6.2 (by Eigenvector Research Inc., WA, USA). In order to handle the sign ambiguity in PARAFAC2, non-negativity constraint is imposed in the *time windows* mode. For IVA, we first performed rank reduction on each frontal slice using the true (or given) number of components, and then used IVA-L-SOS (Bhinge et al., 2019b) to find the demixing matrices. For joint ICA, the third-order tensor is unfolded in the first mode. Following rank reduction of the unfolded data using the given number of components, an ICA algorithm based on entropy bound minimization (ICA-EBM)^2^ (Li and Adali, 2010) is used. We fit every method using multiple random initializations, and use the solution corresponding to the minimum cost value.

#### 2.2.2. Performance Evaluation

We assess the performance of the methods using the following approaches:

- *Factor similarity score*: In order to quantify how well the spatial components extracted by the methods match with ground truth components, we use a similarity score defined as:
  (6) $$\begin{array}{l} {\text{Sim}}_{\text{B}}=\frac{1}{K}\overset{K}{\underset{k=1}{\sum }}\frac{1}{R}\overset{R}{\underset{r=1}{\sum }}{\text{B}}_{k}{\left(:,r\right)}^{T}{\hat{\text{B}}}_{k}\left(:,r\right), \end{array}$$
  where B_*k*_(:, *r*) and ${\hat{\text{B}}}_{k}\left(:,r\right)$ denote the true and estimated *r*th column of the factor matrix in the *voxels* mode corresponding to the *k*th time window, respectively (after fixing the permutation and scaling ambiguity in the methods). Similarly, similarity scores for the first and third mode are computed as follows:
  (7) $$\begin{array}{l} {\text{Sim}}_{\text{A}}=\frac{1}{R}\overset{R}{\underset{r=1}{\sum }}\text{A}{\left(:,r\right)}^{T}\hat{\text{A}}\left(:,r\right),\;{\text{Sim}}_{\text{C}}=\frac{1}{R}\overset{R}{\underset{r=1}{\sum }}\text{C}{\left(:,r\right)}^{T}\hat{\text{C}}\left(:,r\right) \end{array}$$
  Due to different modeling assumptions of each method, all methods can only be compared in terms of Sim_B_. In addition, we report Sim_A_ and Sim_C_ for PARAFAC2, and Sim_A_ for joint ICA.
- *Two-sample* *t-test*: Using two-sample *t*-test on each column of the factor matrix corresponding to the *subjects* mode, i.e., A in PARAFAC2 and joint ICA, and A_*k*_, for *k* = 1, …, *K* in IVA, we identify the statistically significant subject-mode factor vectors in terms of revealing group differences, allowing for unequal variances for healthy and patient groups.

#### 2.2.3. Simulated Data and Experimental Set-Up

**Figure 2:**
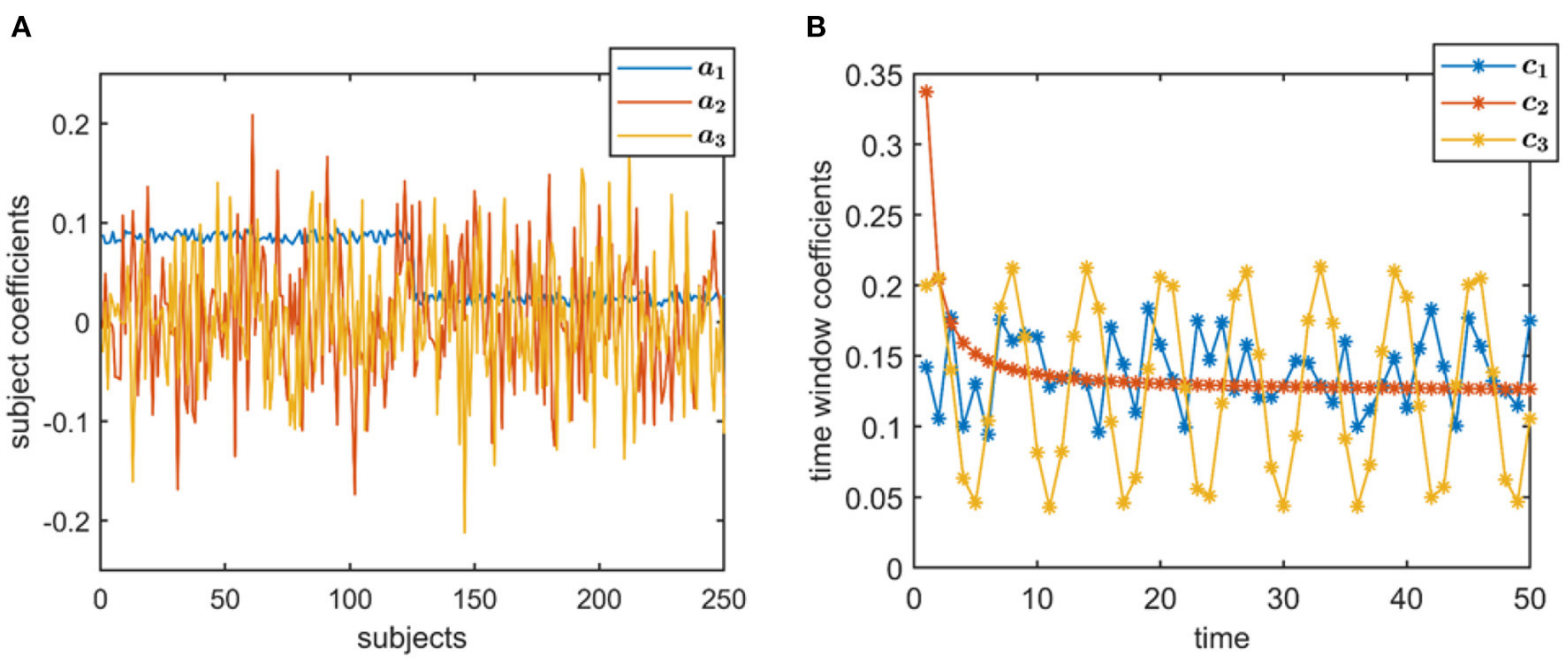
True factors used to generate simulated data. **(A)** Subject-mode factors, where a_*r*_ indicates the columns of A, for *r* = 1, 2, 3, and **(B)** Temporal patterns, where c_*r*_ indicates the columns of C, for *r* = 1, 2, 3. Time, here, is in the resolution of time windows, but may also correspond to time samples depending on the application.

**Figure 3:**
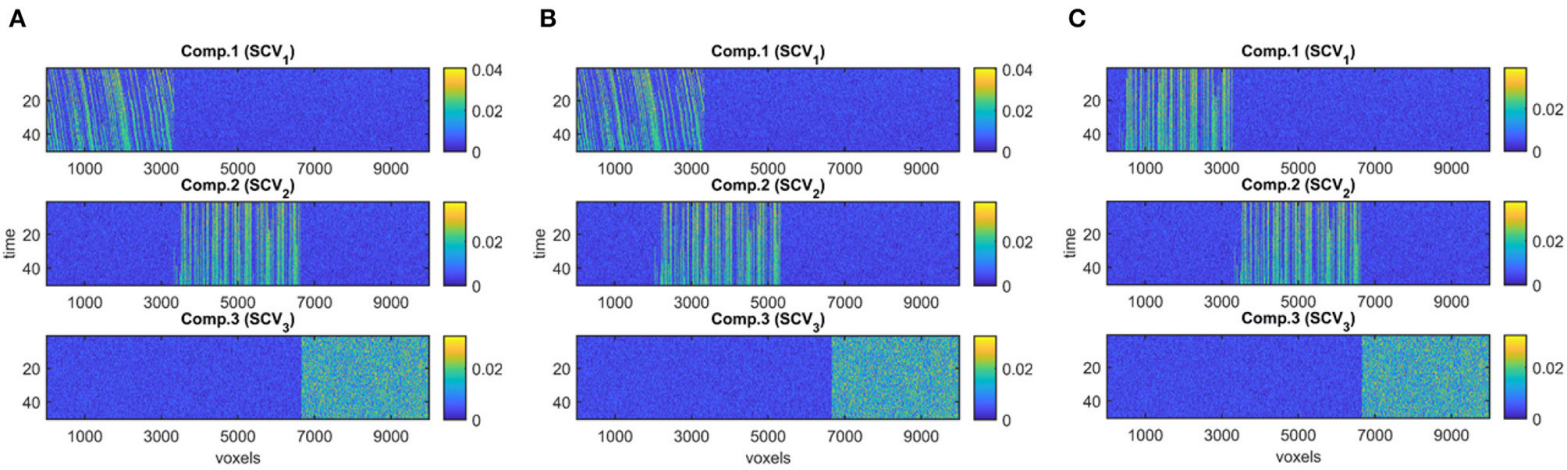
True evolving components (*R* = 3), where each component corresponds to a column of B_*k*_, for *k* = 1, …, 50, used to generate simulated data with **(A)** evolving networks, **(B)** overlapping networks, **(C)** similar networks.

We simulate time-evolving data arranged as a third-order tensor X ∈ ℝ^*I*×*J*×*K*^, with *K* time slices, with the following underlying structure (using *R* = 3 components):

- *Subject-mode patterns*, i.e., A ∈ ℝ^*I*×*R*^, are generated such that one column of A discriminates between two subject groups each containing $\frac{I}{2}$ subjects. Entries corresponding to subjects from different groups are sampled randomly from uniform distributions with different means. Other columns have entries randomly sampled from the standard normal distribution. All columns are normalized to unit norm. The same A with *I* = 250 is used in the experiments (Figure 2A), where the two-sample *t*-test gives the following *p*-values: 0, 0.88, and 0.35.
- *Evolving networks/components* are generated as the columns of ${\text{B}}_{k}\in {\mathbb{R}}^{J\times R}$ (or rows of ${\text{S}}_{k}\in {\mathbb{R}}^{R\times J}$). We generate *R* = 3 evolving networks: The first one is a network that is shifting and increasing in density, the second is increasing in density, and the third one is a random network as shown in Figure 3A. All columns are normalized to unit norm. See the Supplementary Material for more details on the generation of evolving components.
- *Temporal patterns*, i.e., C ∈ ℝ^*K*×*R*^, are generated as (i) a random pattern with uniformly distributed entries, (ii) an exponential decay pattern, and (iii) a pattern following a sinusodial function (see Figure 2B). All columns are normalized to unit norm.

Once factor matrices are generated, the tensor X ∈ ℝ^*I*×*J*×*K*^ is constructed based on (1), and a noisy tensor X_noisy_ is generated as follows:


(8) $$\begin{array}{l} X_{\text{noisy}}=X+\eta N\frac{X_{F}}{N_{F}}, \end{array}$$

where N ∈ ℝ^*I*×*J*×*K*^ has entries randomly drawn from the standard normal distribution, and η indicates the noise level. In the experiments, we use η = 0.5. We use PARAFAC2, IVA, and joint ICA to analyze X_noisy_ using the correct number of components, i.e., *R* = 3, assuming that it is known, and assess their performance in terms of revealing the evolving networks as well as capturing the group difference in the *subjects* mode. We modify the underlying factor matrices for different experimental set-ups of interest and study the relative performance of the methods in the following cases (see the Supplementary Material for additional experiments not specifically focusing on evolving networks):

- *Case 1 (Different sample sizes, different network types):* Here, we study the effect of sample size as well as overlapping and similar networks. In Case 1a, we analyze X_noisy_ generated using different number of dimensions in the *voxels* mode, i.e., *J*, demonstrating the effect of sample size on the performance of the methods. We use *J* = 10, 000 and downsampled versions with a downsampling factor of 20 (i.e., *J* = 500) and 60 (i.e., *J* = 167). Using the same set-up, we also study the effect of the number of time slices, i.e., *K* = 20 and *K* = 50. In Case 1b, with *J* = 10, 000, *K* = 50, we assess the performance of the methods when evolving networks are overlapping as in Figure 3B. Finally, in Case 1c, we consider evolving networks with similar structures, i.e., two of the components are shifting and increasing in density as in Figure 3C. Matrix A and C are as in Figure 2.
- *Case 2 (Different subject-mode matrices):* In this scenario, we study the effect of different subject-mode patterns in different time slices. Each X_*k*_ matrix is constructed using a different ${\text{A}}_{k}\in {\mathbb{R}}^{I\times R}$ matrix in (1). More precisely, ${\text{A}}_{k}=\text{A}+\gamma {\text{N}}_{k}\frac{||{{\text{A}}_{k}||}_{F}}{||{{\text{N}}_{k}||}_{F}}$ for odd values of *k* ≤ *K*, where γ denotes the noise level and is set to γ = 0.3, and ${\text{N}}_{k}\in {\mathbb{R}}^{I\times R}$ is the noise matrix with entries randomly drawn from the standard normal distribution. For even values of *k* ≤ *K*, A_*k*_ are random matrices with entries drawn from the standard normal distribution. This set-up violates assumptions of joint ICA and PARAFAC2 in the subject-mode, and is of interest especially when different subject-mode patterns are possibly expected in different slices, e.g., task vs. rest windows or different tasks. Matrices ${\text{B}}_{k}\in {\mathbb{R}}^{J\times R}$ are as in Figure 3A, downsampled by a factor of 10, i.e., *J* = 1, 000, and matrix C ∈ ℝ^*K*×*R*^ with *K* = 50 is generated in a similar way as in Figure 2B.
- *Case 3 (Strong discriminating component):* Compared with other cases, in this set-up, the main difference is omitting the normalization of the columns of factor matrices A and C resulting in higher 2-norm, i.e., a factor of 4, for the component revealing the group difference. As the evolving components, we use the B_*k*_ matrices in Figure 3A but only the first 15 time slices to match with the number of time slices in real data.

#### 2.2.4. Real fMRI Data

As a real dataset, we analyze images from the MCIC collection (Gollub et al., 2013), a multi-site multi-subject collection of fMRI images from healthy controls and patients with schizophrenia, collected during various tasks. In particular, we use data from the sensory motor (SM) task collected at four research sites: the University of New Mexico, the University of Minnesota, Massachusetts General Hospital, and the University of Iowa. During the SM task, the study participants were equipped with headphones and instructed to listen for sounds of increasing pitch, with a fixation period between each sound. The participants were instructed to press a button whenever they heard a tone. To ensure that conditions were consistent across scan sessions and sites, the MCIC consortium performed meticulous cross-site calibration. For example, the sites had matching button press devices, the intensity of the auditory stimuli were calibrated and the quality assurance procedures recommended by the Biomedical Informatics Research Network for multi-center fMRI studies (Friedman et al., 2006, 2008) were followed.

Based on the blood-oxygenation-level-dependent signal from the SM task, we extracted fALFF (Zou et al., 2008) in sliding time windows, which yields a time-evolving measure of brain activity within each voxel. Note that this approach—using the synchrony across subjects during the task—collapses the time dimension into time windows using fALLF as a feature representing the activity in each time window for each voxel for each subject allowing us to align signals from multiple subjects. The fALFF is calculated by first discarding the high- and low-frequency components to remove noise and signal from the vasculature system. Then, the amplitudes of the frequency components are computed to get the low-frequency fluctuation which is divided by the total amplitude of all frequencies in the time window to obtain the fALFF. To compute the fALFF, we used the REST software v1.8_130615 (Song et al., 2011). We set the window size and stride length to 16 seconds, corresponding to precisely one rest- or task-block in each time window, with no overlap. The low- and high-frequency cutoff for fALFF were set to 0.01 and 0.15, respectively. To construct the data tensor, we used the fALFF values for voxels that correspond to gray matter as feature vectors for each time window and each subject. Each such feature vector has 67,747 elements, leading to a data tensor of size 253 *subjects* by 67,747 *voxels* by 14 *time windows*. No additional preprocessing is carried out to account for site effects (see section 3.2 for more information). Out of 253 subjects, 147 are healthy controls and 106 are patients with schizophrenia.

## 3. Results

Through numerical experiments, we demonstrate that PARAFAC2 and joint ICA capture the underlying networks, their evolution, and reveal the discriminating component accurately irrespective of the sample size as long as the factor matrix in the *subjects* mode stays the same (or differ up to a scaling) across time windows (Case 1 and 3). For these cases, while IVA performs well for large sample size, we often observe that IVA reveals additional components that are statistically significant in terms of group difference in some time windows even though that does not match the ground truth—showing that IVA is more prone to false-positives [i.e., identifying patterns as potentially important (or markers) for group difference] compared to PARAFAC2 and joint ICA. On the other hand, if different time windows have different factor matrices in the *subjects* mode as in Case 2, IVA performs better in terms of revealing the underlying networks. Among the three methods, PARAFAC2 is the only one that reveals compact temporal patterns explicitly.

Our analysis of real task fMRI data demonstrates that all methods (PARAFAC2, IVA, and joint ICA) capture a component including both primary motor, supplementary motor, cerebellum, and temporal regions engaged by the task. This component is also identified as statistically significant in terms of differentiating between healthy controls and patients with schizophrenia. Additional components show up as statistically significant in terms of group difference in IVA in some time windows. However, given the results of our simulations, where we observe small *p*-values for non-discriminating components at some time windows, we discard those components as potential false-positive markers.

### 3.1. Simulations

**Figure 4:**
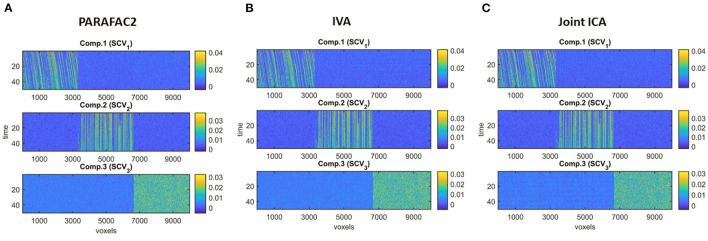
Case 1a. Evolving components (B_*k*_ or S_*k*_, for *k* = 1, …, *K*) captured by each method after fixing the scaling and permutation ambiguity: **(A)** PARAFAC2, **(B)** IVA, **(C)** joint ICA. All methods recover the underlying evolving components accurately.

**Figure 5:**
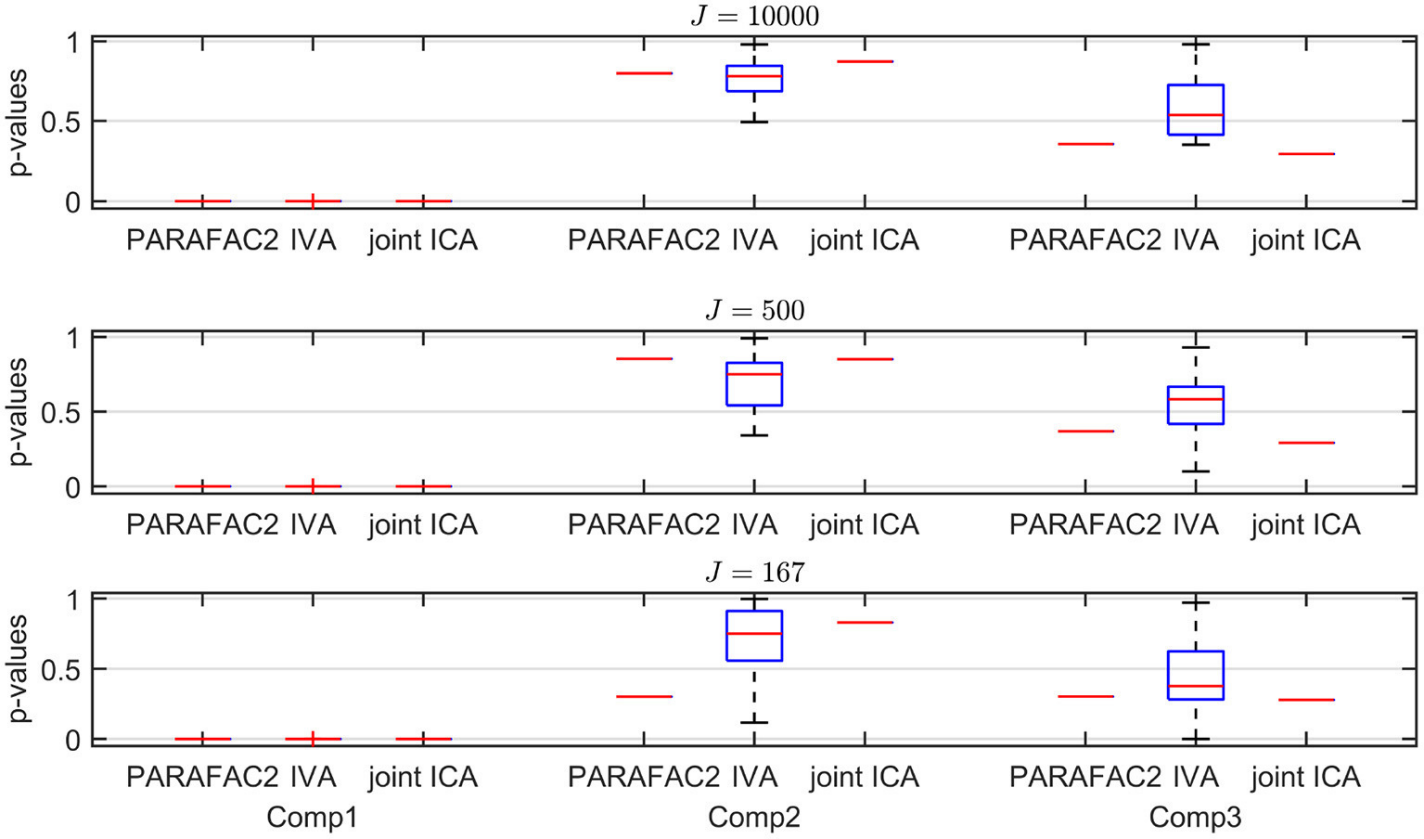
Case 1a (*K* = 20). *p*-values obtained using the two-sample *t*-test on the subject-mode patterns (A or A_*k*_) using different methods as the number of voxels (i.e., *J*) changes, where the number of time slices (i.e., *K*) is set to 20. Based on the true subject-mode patterns, true *p*-values are 0, 0.88, and 0.35 for component 1, 2, and 3, respectively. For large sample size, i.e., *J* = 10, 000, all methods can identify that the first component is the statistically significant one in terms of group difference. As *J* decreases, in addition to the first component, IVA returns small *p*-values for other components in some windows corresponding to false-positive cases while PARAFAC2 and joint ICA work well regardless of the sample size.

Figure 4 demonstrates the evolving components captured by the three methods in Case 1a with *J* = 10, 000 voxels showing that all methods can recover the true underlying evolving networks accurately. Table 1 shows the similarity scores [defined in (6) and (7)] also demonstrating that underlying networks are accurately captured with a similarity score of 1.00 using all methods. Furthermore, all methods perform well in terms of capturing the component discriminating between the subject groups as shown in the top plot in Figure 5. The first component is the one that can separate the two subject groups, with all methods revealing *p*-values around 0, and *p*-values for non-discriminating components are large enough to discard them. Note that since IVA extracts different A_*k*_ matrices, for *k* = 1, …, *K*, different *p*-values are obtained from each matrix and shown as box-plots.

**Table 1:** For each case, dataset sizes (*I, J, K*), similarity scores (Sim_A_, Sim_B_, Sim_C_) showing the accuracy of the methods in terms of capturing the underlying patterns in the first (subject), second (network/voxel), and third (time) modes, respectively, and whether methods give false positive (FP) markers, i.e., identifying components that are not indicating group difference as potential markers with statistically significant group difference.

				**PARAFAC2**				**IVA**		**Joint ICA**		
	I	J	K	**Sim_A_**	**Sim_B_**	**Sim_C_**	**FP**	**Sim_B_**	**FP**	**Sim_A_**	**Sim_B_**	**FP**
Case 1a	250	10,000	50	1.00	1.00	1.00	No	1.00	No	1.00	1.00	No
Case 1a	250	10,000	20	1.00	1.00	1.00	No	1.00	No	1.00	1.00	No
Case 1a	250	500	50	1.00	1.00	1.00	No	1.00	No	1.00	1.00	No
Case 1a	250	500	20	1.00	1.00	1.00	No	1.00	No	1.00	1.00	No
Case 1a	250	167	50	1.00	1.00	1.00	No	1.00	No^*^ (Supplementary Figure 1)	1.00	1.00	No
Case 1a	250	167	20	1.00	0.99	1.00	No	0.99	Yes (Figure 5)	1.00	1.00	No
Case 1b	250	10,000	50	1.00	1.00	1.00	No	1.00	No	1.00	1.00	No
Case 1c	250	10,000	50	1.00	1.00	1.00	No	1.00	No	1.00	1.00	No
Case 2	250	1,000	50	0.99	0.68	0.85	No	0.98	Yes (Figure 8)	0.96	0.74	Yes
Case 3 (*R* = 3)	250	10,000	15	1.00	0.99	1.00	No	0.99	Yes (Figure 9)	1.00	0.99	No
Case 3 (*R* = 4)	250	10,000	15	1.00	0.98	1.00	Yes	0.99	Yes (Supplementary Figure 2)	1.00	0.99	No

* *Indicates that even though there are no false positives, p-values get quite small*.

**Sample Size**. As we decrease the number of samples/voxels (i.e., *J*), we observe differences in the performances of the methods. While all methods can still capture the evolving networks accurately (see Table 1), IVA gets smaller *p*-values even for non-discriminating components in some slices while PARAFAC2 and joint ICA can still clearly identify non-discriminating vs. discriminating components (Figure 5). We observe that the third component also shows up as a statistically significant component in terms of group difference for some time windows using IVA as a false-positive marker. See Supplementary Material also for *K* = 50 (Supplementary Figure 1), where IVA performs better but still returns smaller *p*-values for some components in some windows.

**Figure 6:**
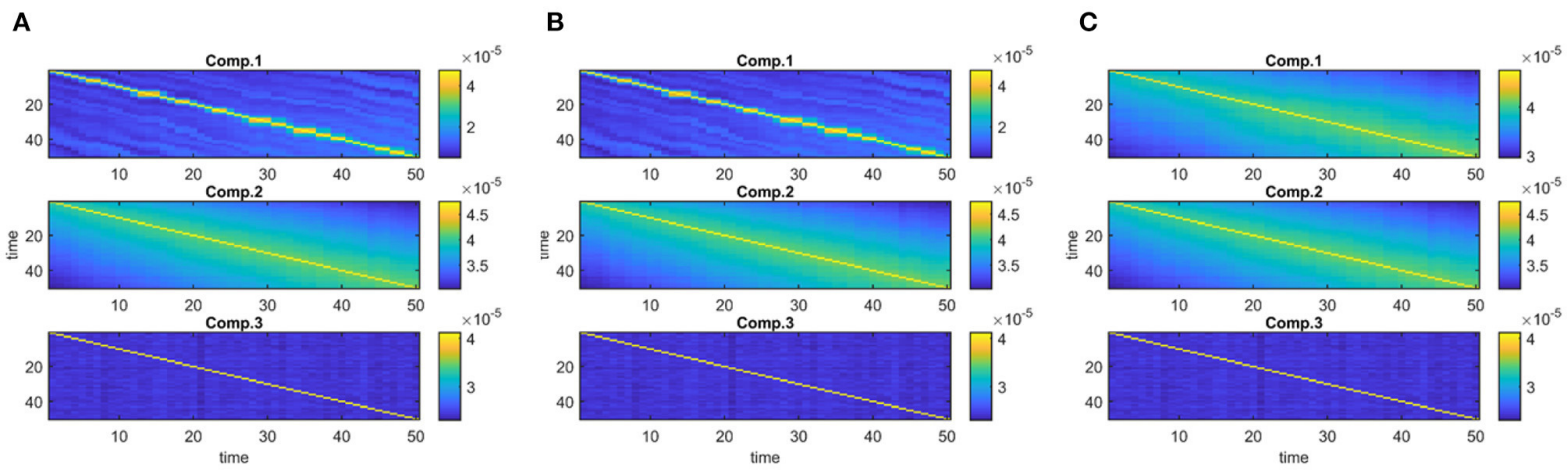
Covariance matrices of size *K* by *K* showing the covariance structure of true components across *K* = 50 time slices for **(A)** evolving networks, **(B)** overlapping networks, **(C)** similar networks.

**Different Network Types**. In the case of different network types, i.e., when we have overlapping evolving components as in Figure 3B, or components evolving in the same way as in Figure 3C resulting in the same covariance structure as in Figure 6C, all methods perform equally well in terms of capturing the underlying components (see the similarity scores in Table 1). The motivation for having overlapping networks is to demonstrate the performance of the methods when networks overlap in space, i.e., *voxels* mode, which may be expected in real applications. Even though the networks overlap, the average correlation of networks, i.e., correlation between columns of B_*k*_ averaged over *K* slices, is small, e.g., ≤ 0.1 in Figure 3B; therefore, not affecting the performance of IVA and joint ICA. Even when there is a larger overlap in space, the correlation is still not high when network structures are different, e.g., shifting vs. non-shifting.

**Figure 7:**
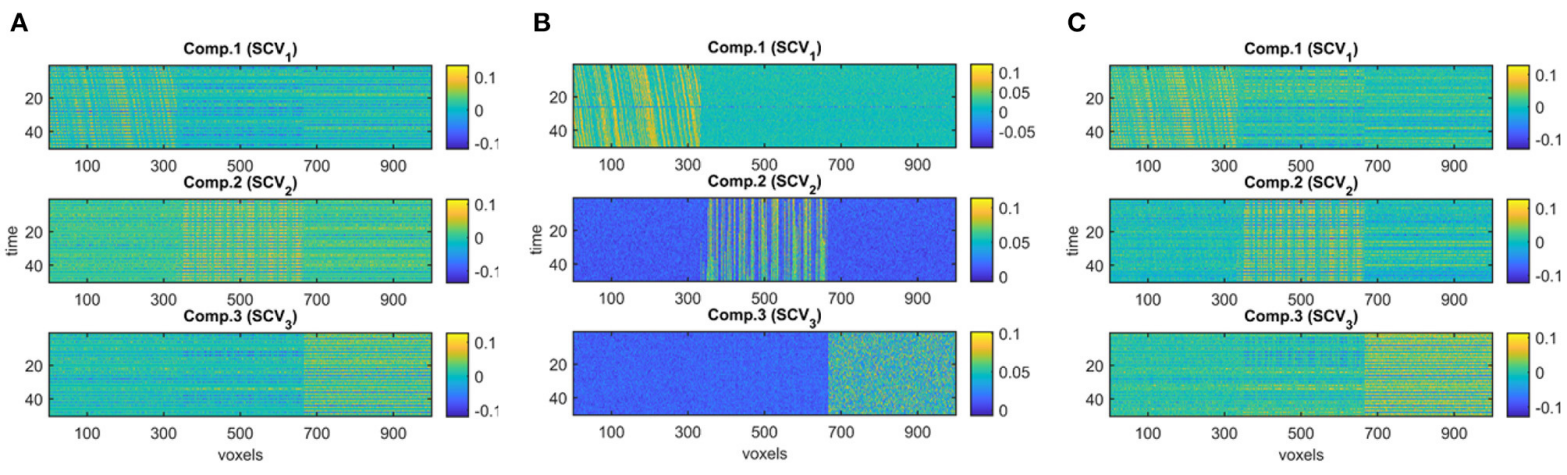
Case 2. Evolving components (B_*k*_ or S_*k*_, for *k* = 1, …, *K*) captured by each method: **(A)** PARAFAC2, **(B)** IVA, **(C)** joint ICA. PARAFAC2 and joint ICA fail to capture the underlying networks while IVA can reveal the evolving components accurately.

**Figure 8:**
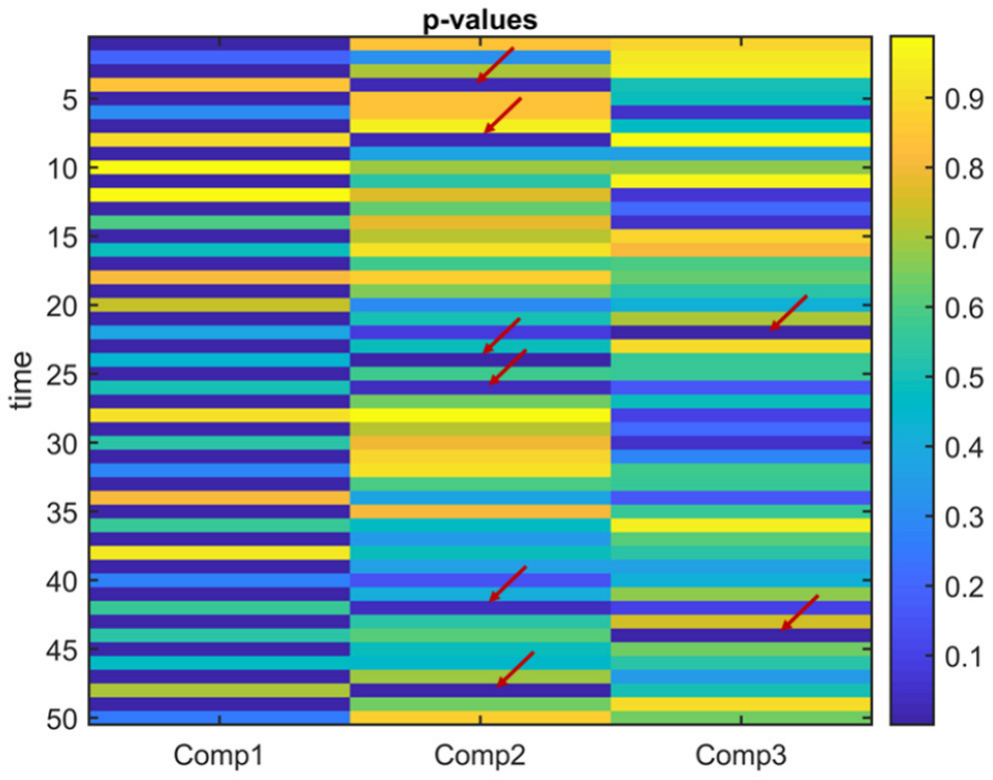
Case 2. *p*-values obtained using the two-sample *t*-test on the subject-mode patterns (A_*k*_) using IVA. IVA successfully captures that the first component is statistically significant in terms of group difference in every other window. In the other components, in some time windows, there are false positives marked with a red arrow.

**Different Subject-Mode Patterns**. When the assumption of the same subject-mode patterns in different time windows is violated, both PARAFAC2 and joint ICA do not capture the underlying evolving components as shown in Figures 7A,C, and with low similarity scores given in Table 1 for Case 2. On the other hand, Figure 7B shows that IVA recovers the evolving components almost accurately with a similarity score of 0.98. Furthermore, IVA also captures that there is a component discriminating between the subject groups in every other window. Figure 8 shows the *p*-values obtained using the A_*k*_ matrices corresponding to each one of the *K* = 50 time slices indicating the statistical significance of the first component in terms of group difference in every other window. For the other components, there are again some small *p*-values as we have also previously observed as a drawback of IVA in Case 1a. Nevertheless, compared to PARAFAC2 and joint ICA, which cannot reveal subject-mode patterns changing from one time slice to another, IVA performs well and can capture such information in one component.

**Figure 9:**
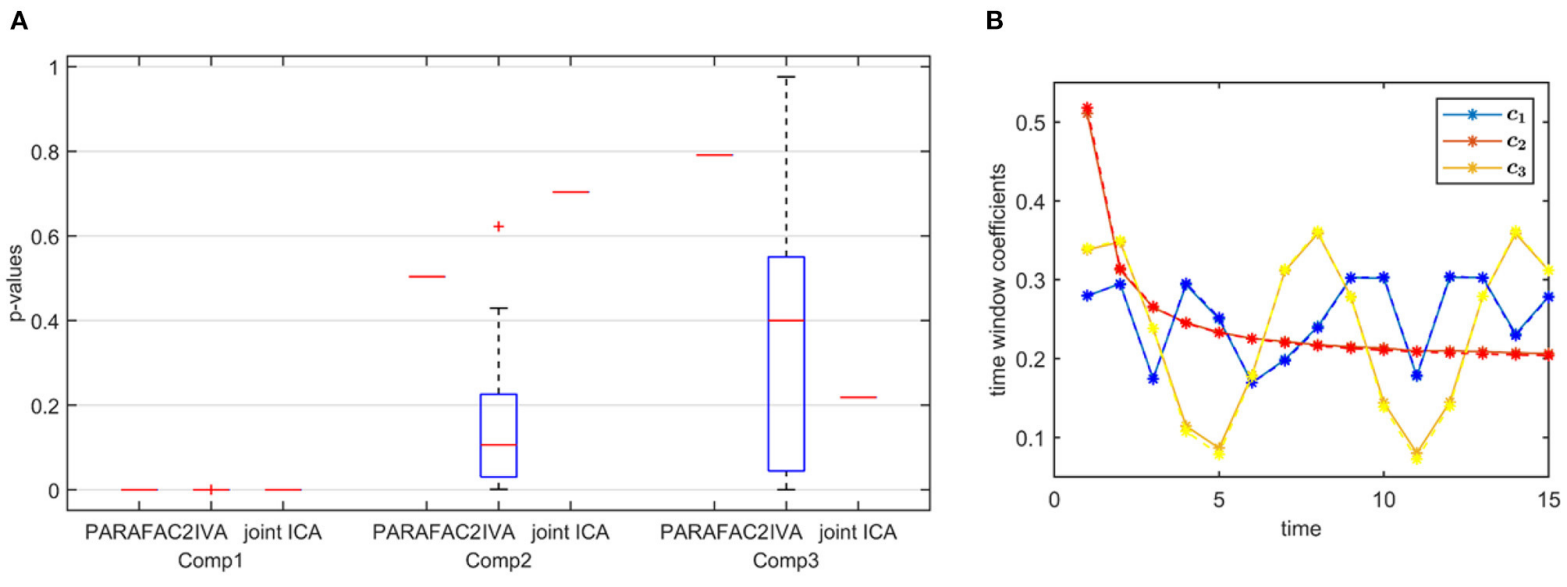
Case 3. **(A)** *p*-values obtained using the two-sample *t*-test on the subject-mode patterns (A or A_*k*_) using different methods. Based on the true subject-mode patterns, true *p*-values are 0, 0.88, and 0.35 for component 1, 2 and 3, respectively. All methods identify the first component as the component differentiating between the subject groups. While PARAFAC2 and joint ICA identify the second and third components as not statistically significant in terms of group difference, IVA wrongly identifies them as statistically significant in some windows. **(B)** Temporal patterns, i.e., columns of factor matrix C, extracted from the *time* mode using PARAFAC2. True patterns are shown using dashed lines. PARAFAC2 correctly captures the true temporal patterns.

**Strong Discriminating Component**. In the presence of a strong component, which is also responsible for the group separation, all methods successfully reveal the underlying evolving components shown by the high similarity scores in Table 1. In the *subjects* mode, Figure 9A demonstrates that PARAFAC2 and joint ICA identify the first component as the discriminating component successfully while IVA has one component that is statistically significant in terms of group difference in all windows and the two other components in some time windows. This set-up is motivated by the real data, where we observe a consistent spatial/voxel-mode pattern using all methods; however, methods differ in terms of subject-mode patterns as a result of their modeling assumptions. PARAFAC2 and joint ICA can reveal the same subject-mode patterns (up to a scaling) in all time windows while IVA may reveal different subject-mode patterns in every time window. In our experiments, we observe that the flexibility of IVA hurts its performance resulting in potentially false-positive markers. Finally, Figure 9B demonstrates the temporal patterns captured by PARAFAC2, revealing the underlying true patterns accurately. Neither joint ICA nor IVA can extract temporal patterns in a compact way. When using IVA, one can focus on how the average subject-mode patterns change from one time window to another (Hossain et al., 2022); however, that relies on the assumption that the average would be representative.

### 3.2. Task fMRI Data Analysis

The fMRI data tensor (constructed as described in section 2.2.4) is in the form of 253 *subjects* by 67,747 *voxels* by 14 *time windows*. Before the analysis, the tensor is preprocessed by subtracting the mean fALFF signal across the *voxels* mode, and dividing each *voxels* mode fiber, i.e., the vector containing the tensor entries for a fixed subject and a time window index, by its standard deviation. The preprocessed tensor is then analyzed using PARAFAC2, IVA and joint ICA in order to capture patterns/networks in the *voxels* mode (as well as their change in time) that can reveal group differences between healthy and patient groups.

**Figure 10:**
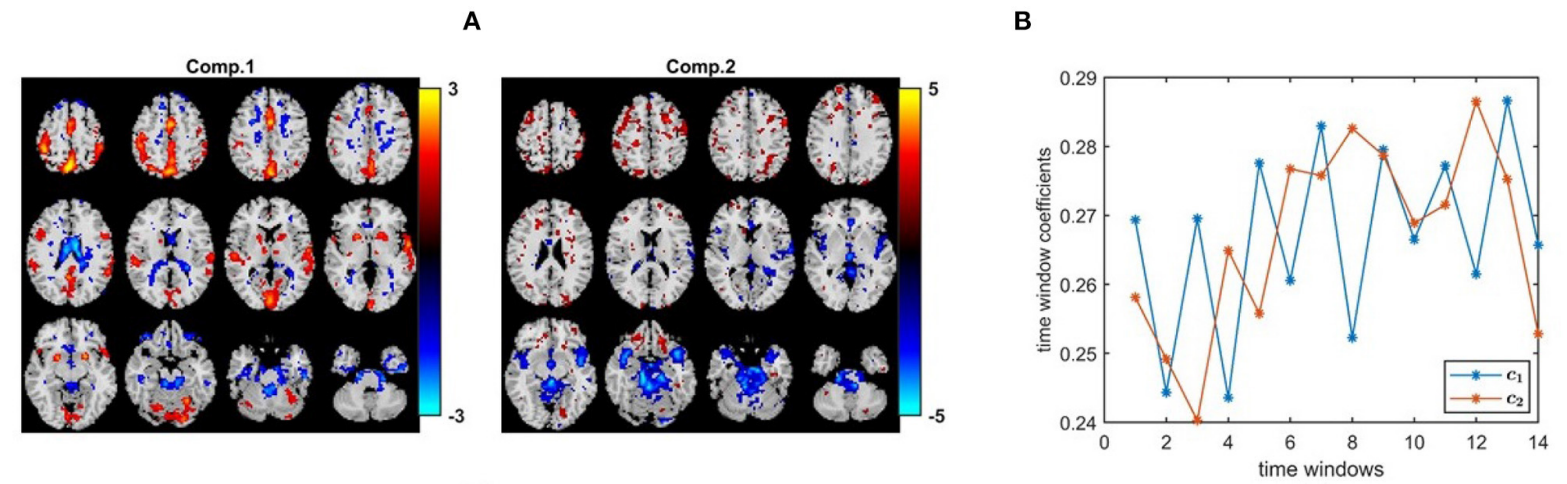
PARAFAC2 analysis of task fMRI data. **(A)** Spatial components, i.e., columns of B_*k*_. Here, we plot columns of only B_1_ corresponding to the first time window. The corresponding *p*-values are 7.8 × 10^−6^ for component 1, and 7.7 × 10^−1^ for component 2. The first component includes primary and secondary motor and cerebellum, as well as auditory cortex expected to be engaged by the task. Spatial maps are plotted using the patterns from the *voxels* mode as z-maps and thresholding at |*z*| ≥ 1.5 such that red voxels indicate an increase in controls over patients, and blue voxels indicate an increase in patients over controls. **(B)** Temporal patterns, i.e., columns of matrix C.

Figure 10A shows the spatial maps captured by a 2-component PARAFAC2 model. These maps correspond to columns of B_*k*_ for the first time window, i.e, *k* = 1. In this article, for all methods, we only show the spatial maps for the first time window. In order to see evolving spatial maps, we refer the reader to the videos in the GitHub repository^3^. The *p*-values are 7.8 × 10^−6^ and 7.7 × 10^−1^ for the first and second component, respectively. The first component is of particular interest since it is statistically significant in terms of group difference. Furthermore, this is a strong discriminating component with a norm that is almost twice the norm of the second component. Importantly, this component includes regions expected to be engaged by the task, e.g., primary and secondary motor and cerebellum, as well as auditory cortex. These regions have also been implicated in schizophrenia (Friston and Frith, 1995; Pearlson and Calhoun, 2007). In Figure 10B, we observe that the first component has a temporal pattern that follows the task-rest pattern. This component is consistently observed when we change the number of components or used data from a subset of the sites. As previously noted, it is challenging to determine the right number of components. We fitted the PARAFAC2 model using *R* = 2, 3, 4 components. While all models had a high core consistency value, i.e., ≥81%, highly correlated factor vectors in the subject-mode were observed using 3-component and 4-component models with the spatial maps in Figure 10A being split into more than one component. Therefore, we focus on the 2-component model, and the fact that the component of interest was also captured as a statistically significant component using a 3-component model gives more confidence in the results we interpret. While we analyze in this article the fMRI data from the four sites available in the MCIC collection (Gollub et al., 2013), in our previous study we only focused on the analysis of two of the sites (Roald et al., 2020) to avoid potential scanner differences and site effects. We observe that despite site effects in the case of four sites, the individual sites show group effects in the same direction relying on the same patterns; therefore we get the same consistent patterns (i.e., spatial maps and the temporal pattern) in both studies confirming that site effects do not have a substantial effect on the patterns of interest.

**Figure 11:**
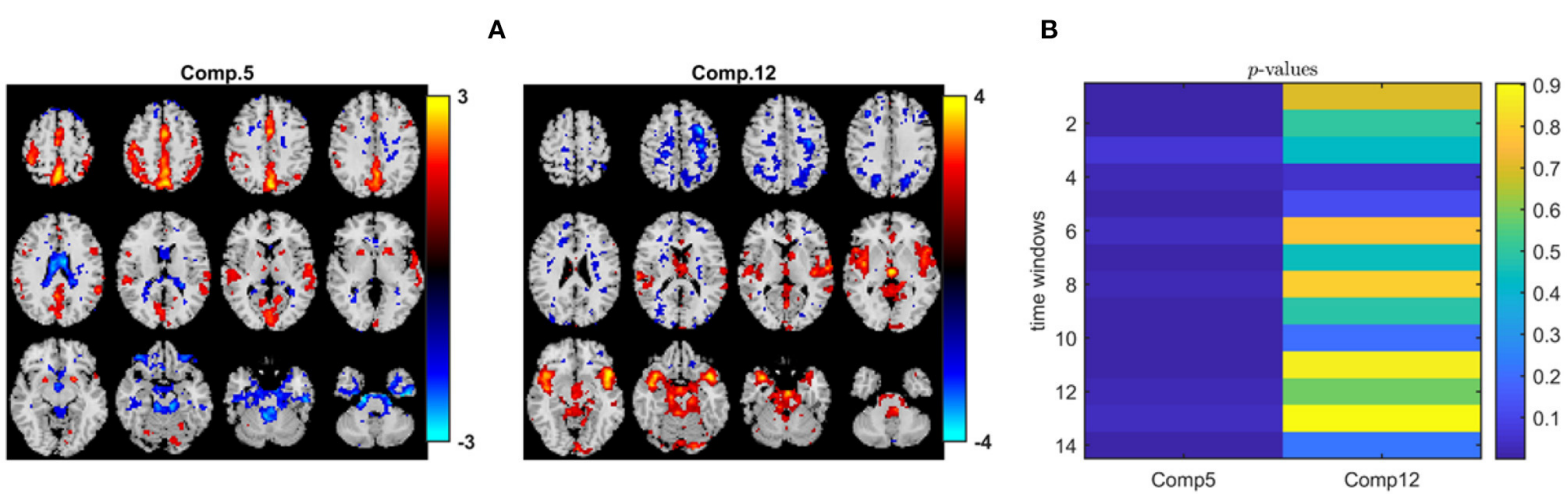
IVA analysis of task fMRI data. **(A)** Spatial components, i.e., rows of S_*k*_. Here, we only plot two of the rows of S_1_ corresponding to the first time window. Spatial maps are plotted using the patterns from the *voxels* mode as z-maps and thresholding at |*z*| ≥ 1.5 such that red voxels indicate an increase in controls over patients, and blue voxels indicate an increase in patients over controls. **(B)** *p*-values for the two components in each time window. While component 5 is statistically significant in all but one time window (i.e., time window 3), component 12 is not in any of the time windows.

When we analyze the data tensor, i.e., multiple matrices in the form of *subjects* by *voxels* matrices corresponding to different time windows, using IVA, we also capture a similar statistically significant sensorimotor component as shown in Figure 11A, i.e., component 5 with activations in the same areas as in component 1 in the PARAFAC2 model (Figure 10A). Since methods have different modeling assumptions, they are not necessarily comparable using the same number of components. We explore a wide range of component numbers to see the performance of the methods using different number of components and compare their best performances. Regardless of the number of components, i.e., *R* = 2, 10, and *R* = 40, IVA reveals this component as a statistically significant component in all but one or two time windows. Here, we report the results using *R* = 40 (see the Supplementary Material for the spatial maps extracted using *R* = 2, which are also very similar to *R* = 40 in terms of the component of interest). Only one out of 40 components is statistically significant in most of the time windows, and that is component 5 in Figure 11A (as also shown in Hossain et al., 2022 on the same dataset). Figure 11B shows that except for one time window, component 5 has a *p*-value ≤ 0.05. Figure 11A shows component 12, which seems to match with the second component in PARAFAC2 (see Figure 10A—Component 2). However, this component is not statistically significant in terms of group difference in PARAFAC2 or IVA^4^.

**Figure 12:**
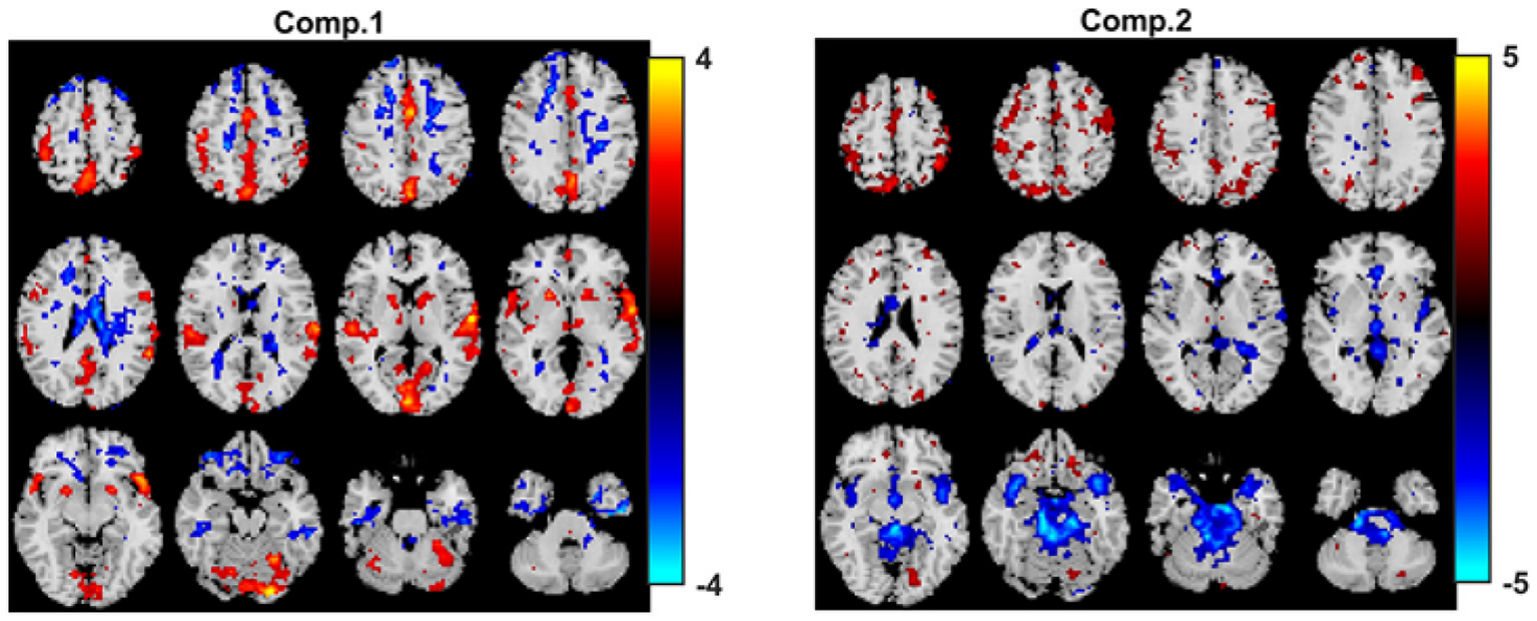
Joint ICA analysis of task fMRI data. Spatial components, i.e., rows of S_*k*_. Here, we only plot rows of S_1_ corresponding to the first time window. Spatial maps are plotted using the patterns from the *voxels* mode as z-maps and thresholding at |*z*| ≥ 1.5 such that red voxels indicate an increase in controls over patients, and blue voxels indicate an increase in patients over controls. The *p*-values are 1.1 × 10^−4^ for component 1, and 1.4 × 10^−1^ for component 2.

When joint ICA is used to analyze the fMRI tensor, as Figure 12 shows, the sensorimotor component is again captured. Here, we include the joint ICA results using *R* = 2 components. The *p*-value for the first component is 1.1 × 10^−4^ while the *p*-value for the second one is ≥0.05. As we increase the number of components (e.g., *R* = 5, 10), joint ICA still reveals the sensorimotor component as the statistically significant one in terms of group difference and no other important component shows up while *p*-values get higher (results not shown). Using higher number of components, e.g., *R* = 40, we observe that the component of interest splits into several components.

Note that while we report *p*-values for comparing how the methods perform in terms of identifying potential components of interest, we do not claim that one is better than the other based on how low the *p*-values are.

**Figure 13:**
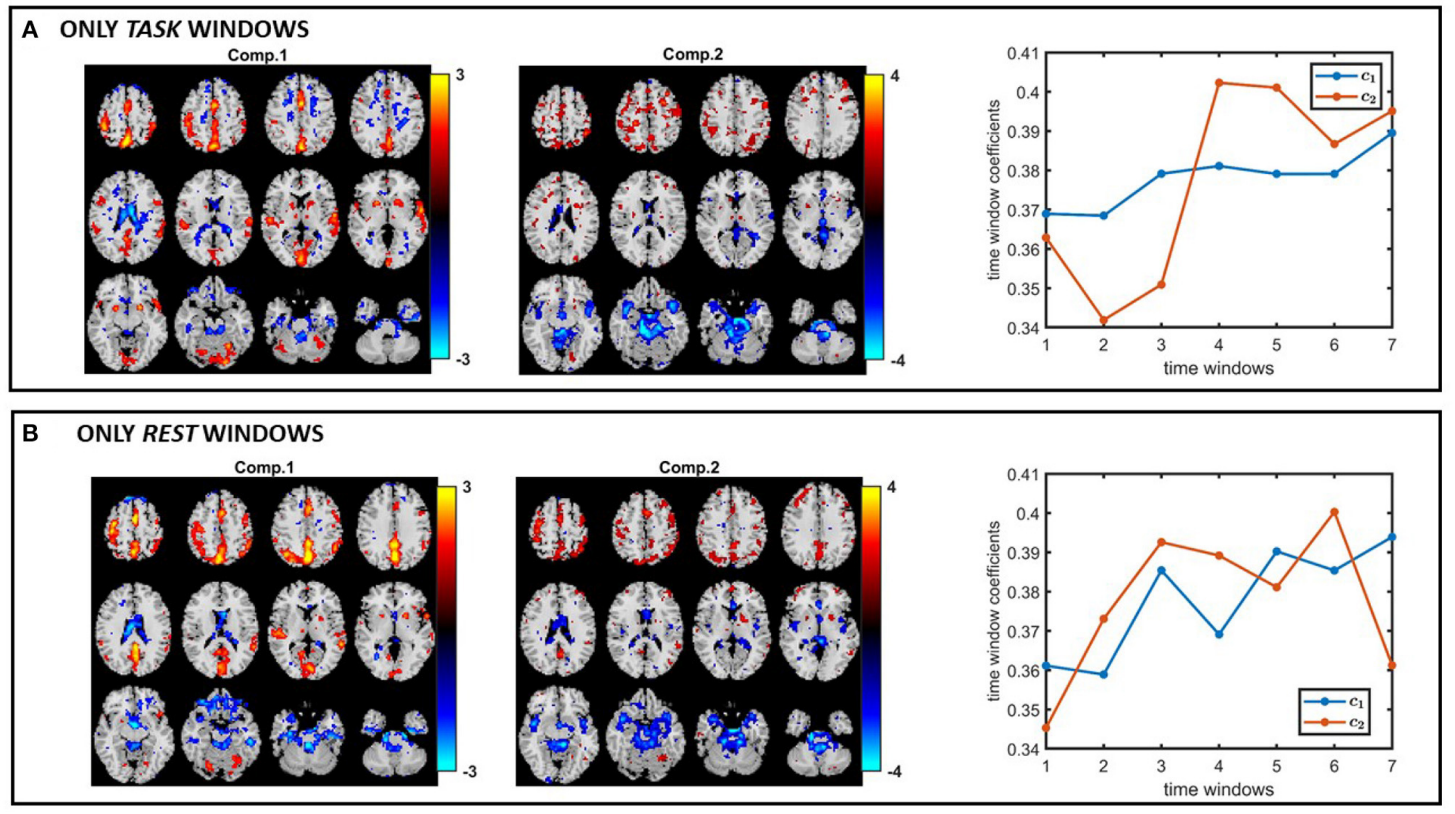
PARAFAC2 analysis of **(A)** only task windows: Spatial components, i.e., columns of B_*k*_, for *k* = 1, as well as the temporal patterns, i.e., columns of C. The *p*-values are 2.1 × 10^−4^ for component 1, and 2.7 × 10^−1^ for component 2. **(B)** Only rest windows: Spatial components, i.e., columns of B_*k*_, for *k* = 1, as well as the temporal patterns, i.e., columns of C. The *p*-values are 6.6 × 10^−3^ for component 1, and 8.5 × 10^−1^ for component 2. The first component shows statistical significance in terms of group difference in both task and rest windows; therefore, supporting the modeling assumptions of PARAFAC2 and joint ICA. Spatial maps are plotted using the patterns from the *voxels* mode as z-maps and thresholding at |*z*| ≥ 1.5 such that red voxels indicate an increase in controls over patients, and blue voxels indicate an increase in patients over controls.

Based on the results of our experiments on simulated data, we know that (i) all methods capture the discriminating component when subject-mode patterns do not change from one time window to another, (ii) IVA often reveals some components that are statistically significant in terms of group difference in few windows—which correspond to false-positive markers. We make the same observations in our real fMRI data analysis. In order to see if the same or similar subject-mode patterns are available in task and rest windows, we analyze only the task windows (i.e., a tensor of size 253 *subjects* by 67,747 *voxels* by 7 *time windows*, for *K* = 1, 3, 5, ..13) as well as only the rest windows (i.e., a tensor of size 253 *subjects* by 67,747 *voxels* by 7 *time windows*, for *K* = 2, 4, ..14). Figures 13A,B show the spatial maps captured using a 2-component PARAFAC2 model from the task tensor and the rest tensor. We observe that the sensorimotor component is statistically significant in terms of group difference in both tensors; therefore, supporting the argument for similar or same subject-mode patterns in different time windows, and making PARAFAC2 and joint ICA suitable approaches for analyzing such time-evolving data.

## 4. Discussion

Overall, all three methods show promising performance in temporal data mining as long as their modeling assumptions are satisfied. Our focus here on addressing the problem of capturing spatial dynamics through the analysis of task fMRI data falls under a temporal data mining problem, where we expect similar group differences (in other words, similar subject-mode patterns) in different time windows. Therefore, PARAFAC2 presents itself as a suitable model providing a compact summary revealing underlying networks, their change in time as well as the temporal patterns in the data. In other scenarios, for instance, when the goal is to analyze multi-task fMRI data, where different slices correspond to fMRI signals collected during different tasks (rather than different time windows), if each task cannot reveal the same group differences, such data may rather follow the modeling assumptions of IVA (Lehmann et al., 2022).

One remaining challenge as a future study is the sensitivity of the methods to the selection of number of components. In simulations, we have assumed that the true number of components is known. While there are various approaches for determining the number of components, often in real applications, the number of components is overestimated. In our real data analysis, we have therefore focused on a component that is consistently observed regardless of the number of components avoiding the sensitivity problem. In order to see the effect of overfactoring, for Case 3, we have fitted PARAFAC2, IVA, and joint ICA using *R* = 4 components, where the true number of components is 3. As shown in Table 1, all methods reveal the evolving networks accurately. However, their performance differs in terms of how well they identify the discriminating component. As we have previously observed in Case 3 when using the true number of components, IVA still identifies additional components as statistically significant in terms of group difference in some slices resulting in many false-positives. In the case of overfactoring, PARAFAC2 also wrongly identifies the additional component as statistically significant in terms of group difference. Joint ICA performs well without any false-positive components. These experiments demonstrate the sensitivity of the methods to the number of components. Note that when the number of component is misspecified, how we select the best run (e.g., the one giving the minimum function value out of multiple initializations) also needs to be studied further, and with the current best run selection approach, the PARAFAC2 model might benefit from regularization in order to prevent overfitting.

There are several other computational aspects that need more research. First, the scalability of the algorithms for fitting the PARAFAC2 model to large-scale data needs to be studied further for dense datasets. The scalability of PARAFAC2 has previously been studied for large-scale sparse data (Perros et al., 2017; Afshar et al., 2018). Another key issue in terms of using PARAFAC2 for time-evolving data analysis is the PARAFAC2 constraint, i.e., constant cross-product constraint. In many applications, that constraint does not have an application-specific justification. We intend to relax the PARAFAC2 constraint, and incorporate additional constraints that will make the analysis time-aware in future studies. While PARAFAC2 ALS algorithm is not flexible enough to incorporate constraints on the evolving patterns, recent work introduces an alternating direction method of multipliers (ADMM)-based algorithm for fitting the PARAFAC2 model enabling imposing constraints in all modes (Roald et al., 2021). It is also worth mentioning that regardless of these advances in computational and modeling aspects of the PARAFAC2 model, the model—as it is—has the potential to reveal time-evolving connectivity patterns if it were to be used in previous connectivity studies assuming static networks (Zhu et al., 2019, 2020).

In this article, we have used different modeling approaches to reveal evolving maps in time and provided them as videos. While such videos show the spatial dynamics to some extent, further work is still needed to quantify and/or better characterize the temporal change from one time window to another—which may be achieved using a postprocessing step or by incorporating relevant constraints into the model.

## 5. Conclusions

Analysis of time-evolving data is challenging especially when the goal is to extract the underlying patterns as well as their evolution. Such analysis is crucial to improve our understanding of complex systems such as the brain. In this article, we study a tensor factorization-based approach called the PARAFAC2 model in comparison with joint ICA and IVA in terms of analyzing time-evolving data and capturing the underlying evolving patterns. Through simulations, we study the performance of these three methods showing that when subject-mode patterns across different time slices are the same, PARAFAC2 and joint ICA perform better in terms of capturing the underlying patterns and are less prone to false-positive markers. On the other hand, if subject-mode patterns differ (more than a scaling factor) from one time window to another, IVA performs the best. In our analysis of real task fMRI data, we observe that all methods capture one consistent component, that is also statistically significant in terms of differentiating between healthy controls and patients with schizophrenia. IVA identifies additional components as statistically significant in terms of group difference; however, those are discarded as potential false positives. Compared to other methods, PARAFAC2 reveals a compact temporal pattern showing the task-rest pattern clearly.

Methods studied in this article are of interest in not only neuroscience but also other fields such as metabolomics to understand the temporal change in human metabolome (i.e., the complete set of small biochemical compounds in the body). For instance, through the analysis of longitudinal metabolomics data as well as data from other sources, it may be possible to capture early signs of diseases (Price et al., 2017). Recently, tensor factorizations have been used to analyze dynamic metabolomics data (Li et al., 2022) but how to capture evolving patterns from such data is yet to be studied.

## Data Availability Statement

The data analyzed in this study is subject to the following licenses/restrictions: While some of the data are available through the COINS (COllaborative Informatics Neuroimaging Suite) database, some of the data are not publicly available (not sharable per the IRB). Requests to access these datasets should be directed to https://coins.trendscenter.org/.

## Ethics Statement

Ethical review and approval was not required for this study as it was deemed ‘not human subjects’ *via* the institutional review board. The patients/participants provided their written informed consent to participate in this study.

## Author Contributions

EA and TA conceived the project and designed the experiments. EA conducted the experiments, with MR being involved in simulated data generation and real data analysis, and KH being involved in real data analysis. EA, MR, TA, and VC were involved in the writing of the manuscript. All authors have given approval to the final version of the manuscript. All authors contributed to the article and approved the submitted version.

## Funding

This work was supported in part by the Research Council of Norway through project 300489 (IKTPLUSS) and by the grants NSF-NCS1631838, NSF-HRD2112455, NIH R01MH118695, and NIH R01MH123610.

## Conflict of Interest

The authors declare that the research was conducted in the absence of any commercial or financial relationships that could be construed as a potential conflict of interest.

## Publisher's Note

All claims expressed in this article are solely those of the authors and do not necessarily represent those of their affiliated organizations, or those of the publisher, the editors and the reviewers. Any product that may be evaluated in this article, or claim that may be made by its manufacturer, is not guaranteed or endorsed by the publisher.

## Footnotes

^1^Both ICA and IVA are traditionally introduced using random variable/vector models. Here to keep the discussion simple across the three models, we use observations for all three models.

^2^The implementation of IVA_L_SOS and ICA_EBM in MATLAB are available at: http://mlsp.umbc.edu/

^3^ https://github.com/eacarat/TracingEvolvingNetworks

^4^The sign of the test statistic is used to fix the sign ambiguity in the models. Both component 2 in PARAFAC2 and component 12 in IVA have a small test statistic; therefore, the uncertainty in the signs and mismatching colors in the maps.
