# Supplementary material for: Tracing Evolving Networks Using Tensor Factorizations vs. ICA-Based Approaches
Source: Front Neurosci. 2022 Apr 25;16:861402. doi: 10.3389/fnins.2022.861402 (PMC9081795; doi:10.3389/fnins.2022.861402)
Supplement: Supplementary file 1 [file Presentation_1.pdf]

frontiers

Supplementary Material: Tracing Evolving Networks using Tensor Factorizations vs. ICA-based Approaches

1 SIMULATIONS

1.1 Generation of Evolving Networks

Evolving components in the simulations are generated as follows: First, voxels are divided into three partitions, one for each component. For the random network component (component 3), we set $\mathbf{B}_k(:,3) = \tilde{\mathbf{B}}_k(:,3)/||\tilde{\mathbf{B}}_k(:,3)||$, where $\tilde{\mathbf{B}}_k(j,3) = \mathbf{L}_k(j,3) + g_{j,3}\mathbf{U}_k(j,3)$ with $\mathbf{L}_k(j,3) \sim \mathcal{N}(0.2, 0.1)$, $\mathbf{U}_k(j,3) \sim \mathcal{U}(0,1)$ and $g_{j,3} = 1$ if voxel j is part of the third voxel-partition and $g_{j,3} = 0$ otherwise. The increasing density and shifting increasing density components (component 1 and 2) are generated as $\mathbf{B}_k(j,r) = \mathbf{L}_k(j,r) + \mathbf{R}_k(j,r)$, where $\mathbf{L}_k(j,r) \sim \mathcal{N}(0.2, 0.1)$ and $\mathbf{R}_k(j,r) \sim \mathcal{N}(0.8, 0.1)$ if voxel j is *active* and $\mathbf{R}_k(j,r) = 0$ otherwise. For the first time step ($k = 1$) for these components, a random subset of the partitioned voxels is selected as active. Then, they increase in density for each time step by adding 15 new activated nodes. The shifting increasing density component (component 1) also has an additional 0.8 chance of all nodes shifting ten indices to the left. Finally, negative entries of the $\mathbf{B}_k$-matrices are set to 0.

1.2 Additional Experiments

While in the main paper, we focus on simulations where the underlying patterns in the *voxels* mode follow evolving network set-up, here we discuss additional simulations demonstrating the underlying assumptions of PARAFAC2, IVA and joint ICA methods. We conduct two sets of experiments:

- *Case 4 - following PARAFAC2 assumptions.*
  Data generation: In Case 4a, $\mathbf{B}_k$ matrices are generated such that they satisfy the constant cross-product constraint, i.e., $\mathbf{B}_k^{\mathsf{T}}\mathbf{B}_k = \mathbf{Q}$, for $k = 1, ..., K$. We follow the same approach as in (Kiers et al., 1999) to generate $\mathbf{B}_k = \mathbf{P}_k\mathbf{B}$, where $\mathbf{P}_k \in \mathbb{R}^{J \times R}$, for $k = 1, ..., K$, is generated as a matrix with orthonormal columns using the QR decomposition of a random matrix, and $\mathbf{B} \in \mathbb{R}^{R \times R}$ is generated through the Cholesky factorization of a positive definite matrix. Matrices $\mathbf{A}$ and $\mathbf{C}$ are the same as in Figure 2 in the paper. In Case 4b, the only difference is $\mathbf{B} = \mathbf{I}$ so that $\mathbf{B}_k$ matrices again satisfy the constant cross-product constraint but in this case, sources/components are orthogonal, i.e., $\mathbf{B}_k^{\mathsf{T}}\mathbf{B}_k = \mathbf{I}$, for $k = 1, ..., K$, where $\mathbf{I}$ is the identity matrix with the appropriate size.
  Results: Figure S3 and Table S1 demonstrate that when data is generated following PARAFAC2 assumptions, PARAFAC2 can find the underlying factors accurately as shown with a similarity score of 1.00 for voxel-mode patterns, and p-values indicating one statistically significant component in terms of group difference and two components with much higher p-values. Joint ICA performs well to some extent but not perfect indicated by a lower similarity score of 0.93 in Table S1. This is a set-up where the sources (i.e., voxel-mode patterns are not statistically independent); therefore, we observe slightly lower performance for joint ICA. IVA, on the other hand, fails to recover the underlying patterns accurately. Even though we give similarity scores and report p-values, this is a case where IVA's uniqueness assumptions are violated, and those metrics do not make sense when the model is not unique. In Case 4b, different than Case 4a, we have orthogonal voxel-mode patterns satisfying joint ICA assumptions. Figure S4 and Table S1 show that both PARAFAC2 and joint ICA capture the underlying factors accurately (as assumptions of both

models are satisfied). On the other hand, IVA performs poorly, and this is again due to lack of uniqueness.

- *Case 5 - different source distributions.*
  Data generation: Here, we generate sources following different distributions (i.e., Laplace vs. Gaussian) in order to show the effect of source distribution in the performance of the methods. In Case 5a, we generate $R = 3$ source component vectors (SCV)s with the covariance structure given in Figure S5 (A), where sources follow a Laplace distribution[2]. In Case 5b, the same set-up with the same covariance structure is used except that sources follow a Gaussian distribution.
  Results: When sources follow a Laplace distribution, all methods perform well. Table S1 and Figure S5 demonstrate that all methods capture the underlying patterns accurately in Case 5a. However, when the sources follow Gaussian distribution, Figure S6 shows that regardless of the algorithmic approach (we use two IVA algorithms, namely IVA-L-SOS the one used in the paper as well as IVA-G (Anderson et al., 2012) assuming Gaussian sources which is a better match for Case 5b), IVA struggles to accurately identify the discriminating component in the *subjects* mode. Figure S6 shows the third component as statistically significant in terms of group difference in some time windows. If the sample size is larger, e.g., $J = 10,000$, IVA achieves to reveal the patterns accurately and identify discriminating components (results not shown). Note that regardless of the source distribution and sample size, both PARAFAC2 and joint ICA accurately find the underlying patterns.

2 SUPPLEMENTARY TABLES AND FIGURES

Table S1. For each case, dataset sizes (I, J, K), similarity scores ($\text{Sim}_{\mathbf{A}}$, $\text{Sim}_{\mathbf{B}}$, $\text{Sim}_{\mathbf{C}}$) showing the accuracy of the methods in terms of capturing the underlying patterns in the first (subject), second (network/voxel), and third (time) modes, respectively, and whether methods give false positive (FP) markers, i.e., identifying components that are not indicating group difference as potential markers with statistically significant group difference.

					PARAFAC2				IVA				joint ICA		
				Source					IVA-L-SOS		IVA-G				
Case	I	J	K	Distr.	$\text{Sim}_{\mathbf{A}}$	$\text{Sim}_{\mathbf{B}}$	$\text{Sim}_{\mathbf{C}}$	FP	$\text{Sim}_{\mathbf{B}}$	FP	$\text{Sim}_{\mathbf{B}}$	FP	$\text{Sim}_{\mathbf{A}}$	$\text{Sim}_{\mathbf{B}}$	FP
4a	250	1000	20	Gaussian	1.00	1.00	1.00	no	0.79	yes	-	-	0.96	0.93	no
4b	250	1000	20	Gaussian	1.00	1.00	1.00	no	0.83	yes	-	-	1.00	1.00	no
5a	250	1000	20	Laplace	1.00	1.00	1.00	no	1.00	no	-	-	1.00	1.00	no
5b	250	1000	20	Gaussian	1.00	0.99	1.00	no	0.96	yes	0.98	yes	1.00	0.99	no

REFERENCES

Anderson, M., Adali, T., and Li, X.-L. (2012). Joint blind source separation with multivariate gaussian model: Algorithms and performance analysis. *IEEE Transactions on Signal Processing* 60, 1672–1683

Kiers, H. A., Ten Berge, J. M., and Bro, R. (1999). PARAFAC2 - part i. a direct fitting algorithm for the PARAFAC2 model. *Journal of Chemometrics* 13, 275–294

[2] We use MGGD_generation.m function from `http://mlsp.umbc.edu/resources.html`

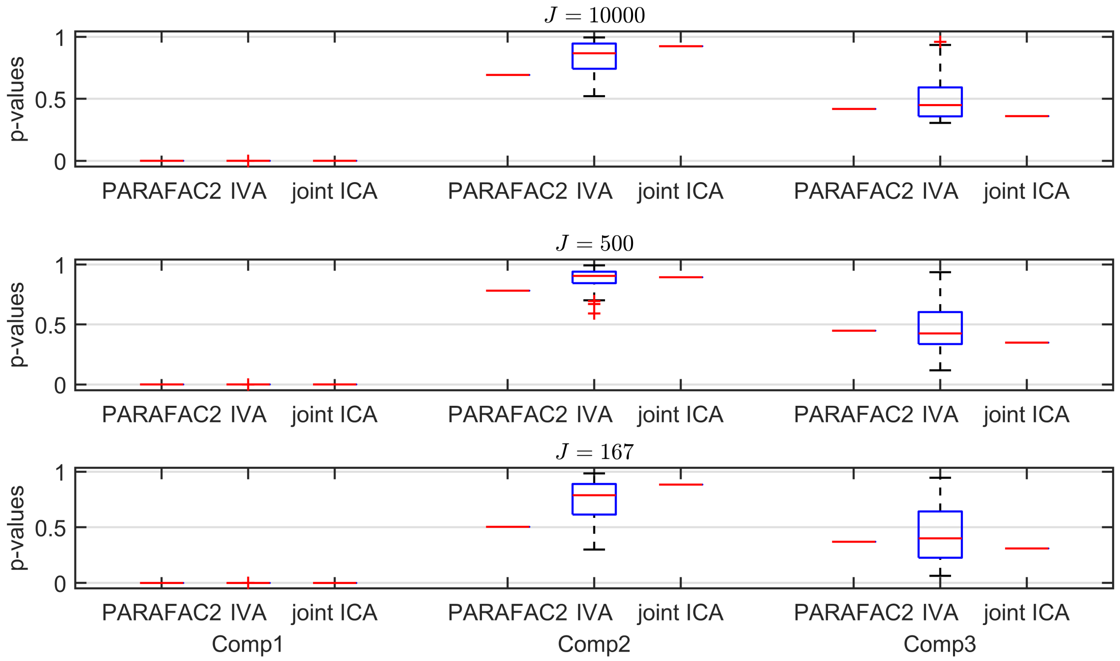

Figure S1: *Case 1a ($K = 50$).* p-values obtained using the two-sample t-test on the subject-mode patterns ($\mathbf{A}$ or $\mathbf{A}_k$) using different methods as the number of voxels (i.e., J) changes, where the number of time slices (i.e., K) is set to 50. Based on the true subject-mode patterns, true p-values are 0, 0.88 and 0.35 for component 1, 2 and 3, respectively. For large sample size, i.e., $J = 10,000$, all methods can identify that the first component is the statistically significant one in terms of group difference. As J decreases, in addition to the first component, IVA returns small p-values for other components in some windows while PARAFAC2 and joint ICA clearly work well regardless of the sample size.

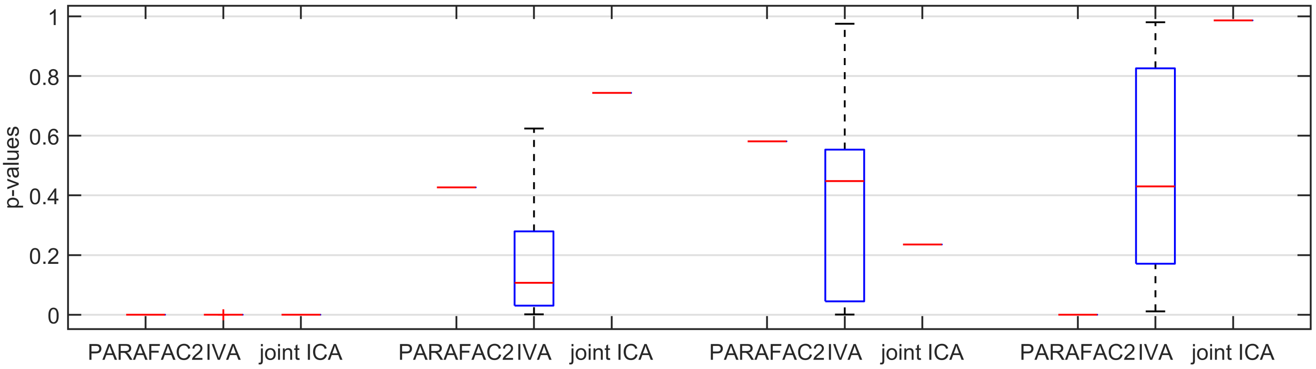

Figure S2: *Case 3 ($R = R_{true} + 1$).* p-values obtained using the two-sample t-test on the subject-mode patterns ($\mathbf{A}$ or $\mathbf{A}_k$) using different methods. Methods are fitting using R components, where the true number of components is $R_{true} = 3$. Based on the true subject-mode patterns, true p-values are 0, 0.88 and 0.35 for component 1, 2 and 3, respectively. All methods identify the first component as the discriminating one successfully. However, IVA returns small p-values for other components in some windows as well, PARAFAC2 wrongly identifies the additional component as statistically significant while joint ICA works well.

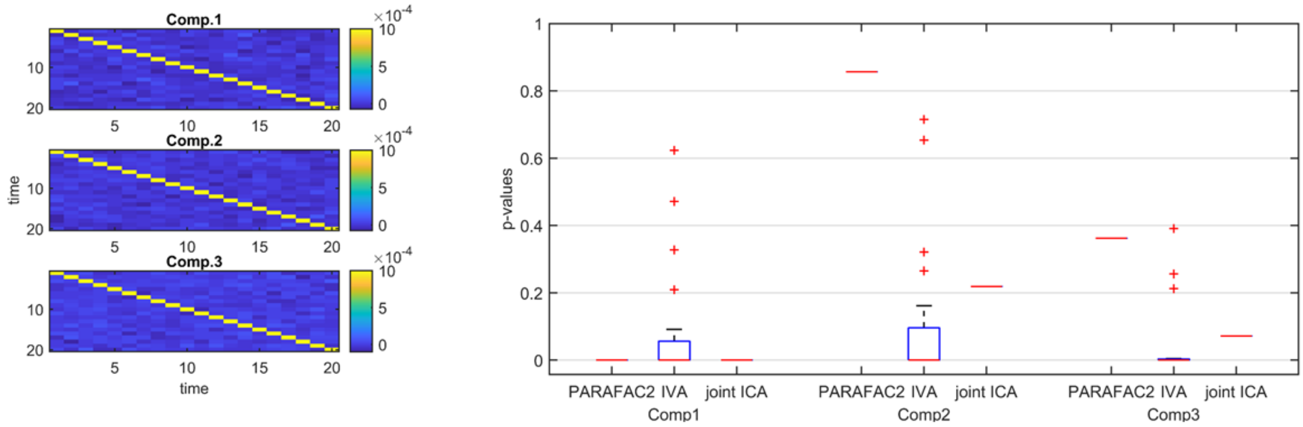

(A) (B)

Figure S3: *Case 4a.* (A) Covariance matrix of size K by K showing the covariance structure of each component across $K = 20$ slices, (B) p-values obtained using the two-sample t-test on the subject-mode patterns ($\mathbf{A}$ or $\mathbf{A}_k$) using different methods. Based on the true subject-mode patterns, true p-values are 0, 0.88 and 0.35 for component 1, 2 and 3, respectively. PARAFAC2 performs well by identifying only the first component as the discriminating component, IVA fails due to non-uniqueness, and joint ICA identifies the discriminating component but the components do not perfectly match with the ground truth (Table S1).

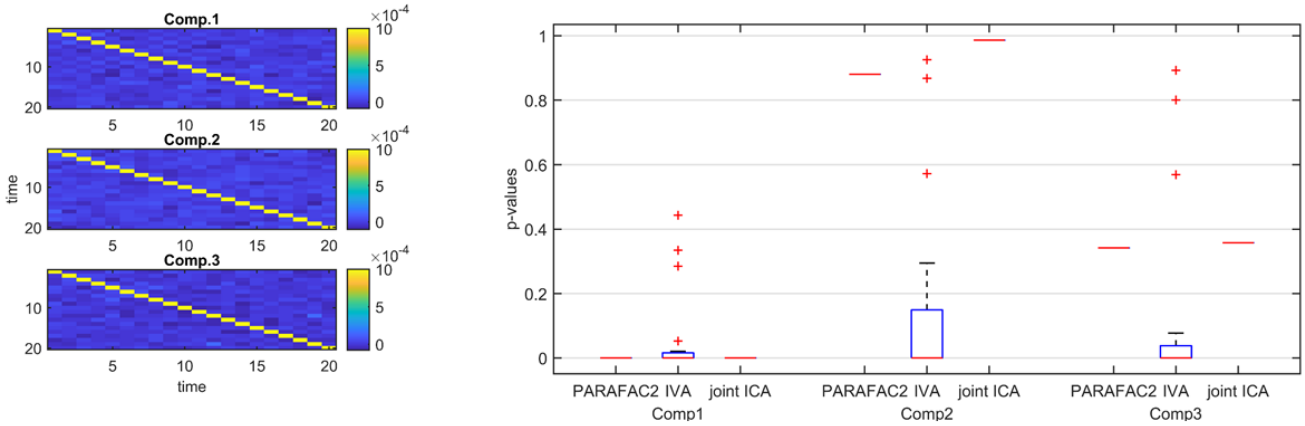

(A) (B)

Figure S4: *Case 4b.* (A) Covariance matrix of size K by K showing the covariance structure of each component across $K = 20$ slices, (B) p-values obtained using the two-sample t-test on the subject-mode patterns ($\mathbf{A}$ or $\mathbf{A}_k$) using different methods. Based on the true subject-mode patterns, true p-values are 0, 0.88 and 0.35 for component 1, 2 and 3, respectively. Both PARAFAC2 and joint ICA can identify the first component as the discriminating one and the other components as not statistically significant in terms of group difference. IVA, on the other hand, fails due to uniqueness issues.

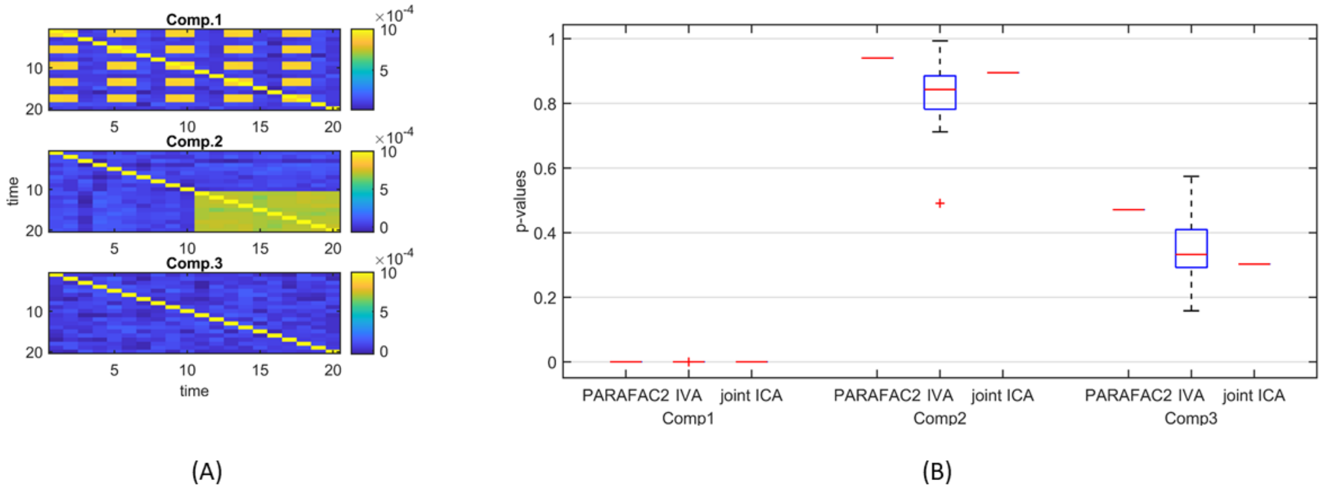

Figure S5: *Case 5a.* (A) Covariance matrix of size K by K showing the covariance structure of each component across $K = 20$ slices, (B) p-values obtained using the two-sample t-test on the subject-mode patterns ($\mathbf{A}$ or $\mathbf{A}_k$) using different methods. Based on the true subject-mode patterns, true p-values are 0, 0.88 and 0.35 for component 1, 2 and 3, respectively. All methods accurately identify discriminating vs. non-discriminating components.

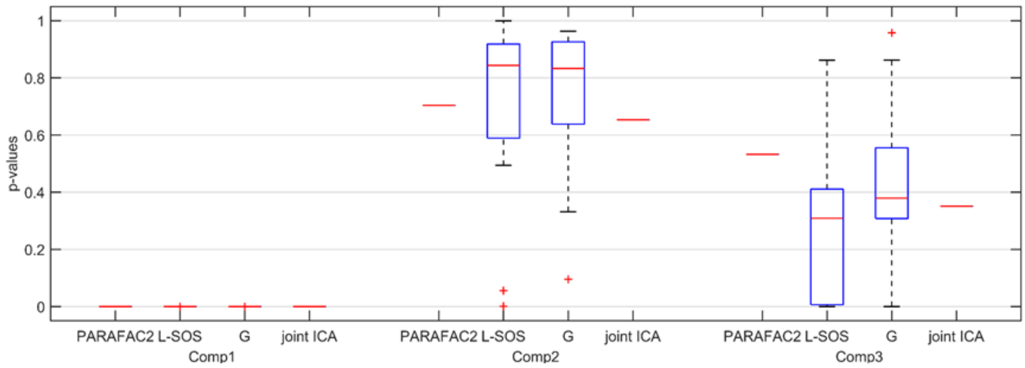

Figure S6: *Case 5b.* p-values obtained using the two-sample t-test on the subject-mode patterns ($\mathbf{A}$ or $\mathbf{A}_k$) using different methods. L-SOS and G stand for IVA-L-SOS and IVA-G, respectively. Sources follow Gaussian distribution, and $J = 1000$. Based on the true subject-mode patterns, true p-values are 0, 0.88 and 0.35 for component 1, 2 and 3, respectively. Regardless of the algorithmic approach, IVA wrongly identifies component 2 and 3 as statistically significant in terms of group difference in some windows while PARAFAC2 and joint ICA work well by only identifying the first component as the discriminating component.

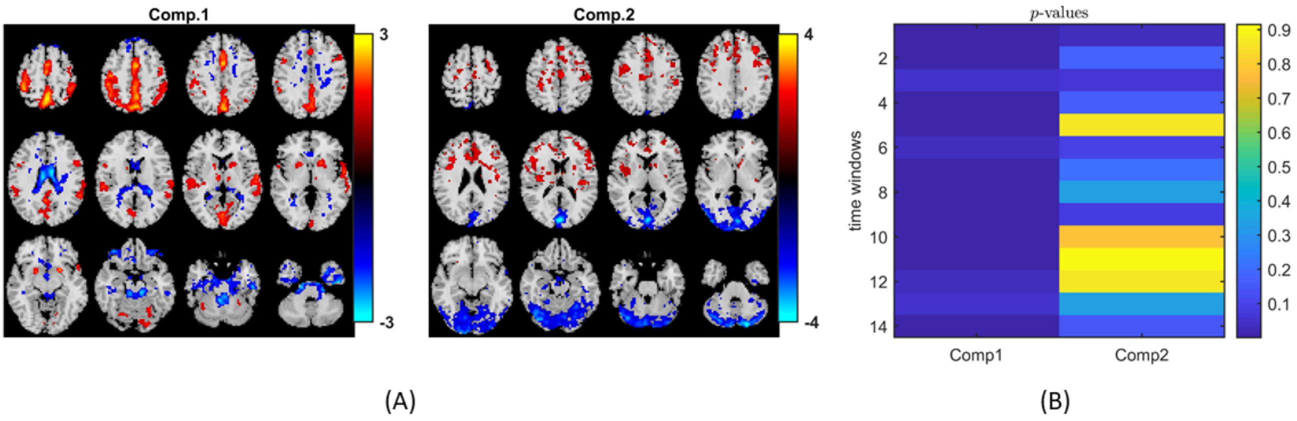

Figure S7: *IVA analysis of task fMRI data using* $R = 2$ *components.* (A) Spatial components, i.e., rows of $\mathbf{S}_k$. Here, we only plot the rows of $\mathbf{S}_1$ corresponding to the first time window. Spatial maps are plotted using the patterns from the *voxels* mode as z-maps and thresholding at $|z| \geq 1.5$ such that red voxels indicate an increase in controls over patients, and blue voxels indicate an increase in patients over controls, (B) p-values of the components in each time window. Component 1, which matches with the sensorimotor component of interest, is statistically significant in all but two time windows while p-values for the second component are ≥ 0.5 in all windows except one.
